# Human foetal cortical slice cultures: A translational platform for studying early neurodevelopment

**DOI:** 10.1111/joa.70276

**Published:** 2026-11-14

**Authors:** Faye McLeod, Anna Dimtsi, Michael A. Savage, Ann Hedley, Gavin J. Clowry, Andrew J. Trevelyan

**Affiliations:** ^1^ Biosciences Institute and Centre for Transformative Neuroscience Newcastle University Newcastle upon Tyne UK; ^2^ Bioinformatics Support Unit Newcastle University Newcastle upon Tyne UK

**Keywords:** calcium imaging, human organotypic slices, neurodevelopment, neuronal network activity, subplate neurons, transcriptomics

## Abstract

The developing human neocortex is characterised by complex and dynamic changes in cellular architecture, neuronal organisation and activity. We previously described how human foetal cortical organotypic slice cultures, prepared from ethically sourced tissue could be used to investigate these processes. Cultures can be maintained for weeks, exhibiting features of the normal developing brain that are not replicated in current organoid models. Such cultures allow the possibility for experimental manipulation, for instance testing the effects of genetic mutations. Here we extend the characterisation of our model. We report that these organotypic cultures preserve the laminar organisation of the developing cortical wall and show considerable diversity of neuronal morphology. Employing bulk mRNA hybridisation for 794 genes (a “human neuropathology” gene panel), we identified consistent patterns of evolving gene expression between 0 and 14 days in vitro (DIV) across different tissue samples taken at 16–18 post‐conceptional weeks (pcw). To put this in the context of the normal developmental pattern, we compared our data set with previously acquired tissue RNAseq data collected between 7–17 pcw (https://www.hdbr.org/expression). This indicated that many gene expression changes in vitro followed prior developmental trajectories, but others diverged strongly, including upregulated genes associated with responses to stress, and downregulated genes associated with the vascular function. This latter effect may reflect the redundancy of vascular development in culture. Notably, various genes associated with pre‐ and post‐ synaptic function, including both glutamate and GABA receptors, continued to be expressed strongly. Consistent with this, we observed spontaneous neuronal network activity in the subplate (SP) using targeted expression of the genetically encoded Ca^2+^ indicator, GCaMPf6, to post‐mitotic neurons. The frequency of neuronal Ca^2+^ transients increased when blockers of either GABA_A_ receptors or gap junctions were applied. Conversely, activity was significantly reduced by glutamatergic receptor antagonists and abolished by voltage gated sodium channel blockade. Neuronal Ca^2+^ transients were triggered directly by focal application of glutamate in the SP and subventricular zone, whereas cortical plate neurons exhibited weaker responses. This was consistent with levels of immunoreactivity for glutamate receptor sub‐units in these compartments. In conclusion, the organotypic cultures show functional synaptic networks, supported by continued expression of the relevant synaptic protein machinery. These data provide further validation of organotypic cultures as a model for investigating and manipulating the early prenatal development of human neocortex, a period that is otherwise difficult to study.

## Graphical abstract

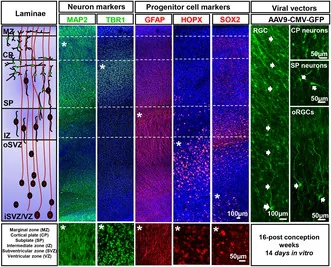

## 1 INTRODUCTION

There are various model systems for investigating early brain development and associated neurological disorders, each offering different benefits and drawbacks. Animal models enable both in vivo and in vitro investigations, including behavioural studies, disease symptoms, brain electrophysiology, and biochemical assays. However, critical differences between rodents and humans, such as in homologue gene expression, brain volume and structure, can present problems for the clinical translation of these results (Azevedo et al., 2009; Herculano‐Houzel et al., 2006; Milior et al., 2023). Human derived induced pluripotent stem cells (iPSCs) provide an alternative platform; they can be differentiated into specific cell types and have been widely used to study several neurodevelopmental disorders at the cellular level (Abud et al., 2017; Chambers et al., 2009; Madhavan et al., 2018), but these models lack the macroscopic organisation of brains. To overcome this issue, organoids, derived from stem cells, and which include neurons together with oligodendrocytes, astrocytes, microglia, and vascular structures, provide model systems with increased cell diversity and some of the macroscopic structure of the brain (Ham et al., 2020; Lancaster et al., 2013; Sloan et al., 2017). Different region‐specific organoids fused in culture (assembloids, (Birey et al., 2017)) or combinations of different iPSC‐derived cell types (Workman et al., 2017) have been used to study cell migration, and cellular and cross‐regional interactions.

Organoids and assembloids do have certain notable limitations, however, such as the absence of any direct homolog of the subplate (SP; (Andrews & Kriegstein, 2022, Di Lullo & Kriegstein, 2017, Eichmuller & Knoblich, 2022, Qian et al., 2019)), a transient structure which is considered to play a crucial role in the development of the cerebral cortex (Molnar et al., 2020; Ohtaka‐Maruyama, 2020). In utero, the earliest precursor of neocortex is the preplate, which splits to form the marginal zone (MZ) at the pial surface, and the presubplate (preSP) deep to the cortical plate (CP), at around 7.5 pcw in humans (Bystron et al., 2008; Meyer et al., 2000). The CP will go on to form layers 2–6 of the cortical grey matter. By 13 pcw, the presubplate condenses with the lower part of layer 6 to form the SP proper (Bayatti et al., 2008; Duque et al., 2016; Kostovic & Rakic, 1990), which contains both younger migrating neurons and established (post‐migratory) neurons. Two weeks later, the SP is nearly twice the width of the CP, with a low density of neurons arranged in distinct superficial and deep layers (but with a high density of axons and synaptic neuropil (Kostovic et al., 2019, Wang et al., 2010). Indeed, the SP, along with the MZ, is the earliest site of synapse formation in the developing cortex, with innervation by thalamocortical axons appearing as early as 8 pcw (Alzu'bi et al., 2019). By 8–10 pcw, synapses have been seen in both the MZ and preSP using immunohistochemistry and electon microscopy (Bayatti et al., 2008; Kostovic & Rakic, 1990). The SP, itself, appears around 13 pcw, and contains most of the earliest neurons to be born; these early developmental milestones in the SP suggest that it is likely to play a vital role in integrating spontaneous and sensory driven activity patterns that sculpt the functional neuronal networks of the mature cortex (Molnar et al., 2020; Mukherjee & Kanold, 2022). The SP persists until around term, after which it largely disappears, although small numbers of SP neurons identified by certain markers (e.g. CPLX3 and NURR1) have been observed to survive in the adult cortex, in mice, particularly in layers 1 and 6 (Hoerder‐Suabedissen & Molnar, 2013; Zolnik et al., 2024).

Its ephemeral nature, and the fact that this coincides almost exactly with the in utero period, present serious practical difficulties for studying SP development. Nor are cerebral organoids or animal models suited for this task. Organoids form electrically active networks of certain classes of neuron that are found in vivo (Fair et al., 2020; Qian et al., 2020), but they lack other SP cell classes, and also differ markedly with respect to its macroscopic structure (Ham et al., 2020; Lancaster et al., 2013; Sloan et al., 2017). Rodent models are similarly limited; the rodent SP consists of a thin layer of cells and does not increase in size during development (Wang et al., 2010).

To address this shortfall, we previously established a human organotypic slice culture model derived from ethically sourced foetal cortex tissue at 14–18 pcw (McLeod et al., 2023, 2026). This model preserved the cortical wall architecture, including the SP lamina, which appeared rich in the pre‐synaptic proteins synapsin1 and STXBP1. Markers of glutamatergic (vGlut1, HOMER1) and GABAergic (vGAT, Gephyrin) synapses, as well as the pre‐synaptic marker STXBP1, increased over 4 weeks in vitro, indicative of neuronal network maturation in this location. Crucially, this chronic culture model is amenable to genetic manipulation such as knocking down gene expression with short hairpin RNA (shRNA) or introducing biosensors such as Ca^2+^ indicators (McLeod et al., 2023, 2026). Our earlier work focused upon the effects of knocking down STXBP1 expression. Here, we continue the characterisation of the baseline model system without gene manipulation, focusing in particular upon the cellular composition and gene expression profile at the time of tissue acquisition and how these change over time in vitro. Additionally, we examine the nature of the spontaneous neuronal activity in these cultures.

## 2 MATERIALS AND METHODS

### 2.1 Human organotypic slice cultures

Human foetal brain tissue was ethically sourced from the joint MRC/Wellcome Trust funded Human Developmental Biology Resource (HDBR, https://www.hdbr.org/ Project 200428), with appropriate maternal consent. Ethical approval was granted from the Newcastle and North Tyneside NHS Health Authority Joint Ethics Committee (REC reference: 18/NE/0135). Cultures were prepared from six different human foetal donor brains aged 16–18 pcw (Table 1), as described previously (McLeod et al., 2023). In brief, 280–300 μm thick coronal sections of occipital cortex were prepared using a 5100 mz Vibratome (Camden Instruments), and these were transferred to individual wells, in 12‐transwell plates, and bathed with BrainPhys (5790; Stemcell Technologies) culture media with added 1 × N2, 1 × B27, 40 ng/mL Brain‐derived Neutrophic Factor (BDNF), 20 ng/mL Glia‐derived Neurotrophic Factor (GDNF), 30 ng/mL Wnt7a, 200 nm ascorbic acid, 1 mm dibutyryl cyclic AMP and 1 μg/mL laminin. All cultures were maintained at 37°C, 5% CO_2_ in ambient O_2_ and 90% humidity. The culture media was changed every 3 days.

**TABLE 1:** List of human donors.

Age (pcw)	Brain region	Experiment (figure number)
16	Occipital	1, 2, 6
17	Occipital	3–6
17	Occipital	3–6
17	Occipital	3–5
18	Frontal	6–10
16	Occipital	6–10

### 2.2 Viral vectors

To introduce fluorescent markers, two adeno‐associated viral (AAV) vectors were used:

1. GCaMP6f—packaged in an AAV9 vector expressing the neuron specific Synapsin promoter to achieve a final titre of 1. × 10^12^ GC/mL. This virus was purchased from Addgene (100836‐AAV9). GCaMP6f is a genetically encoded calcium indicator used to monitor changes in intracellular calcium levels.
2. AAV9‐CMV‐GFP—an AAV9 vector expressing green fluorescent protein (GFP) under the control of the ubiquitous cytomegalovirus (CMV) promoter, with a final titre of 1 × 10^13^ GC/mL. This virus was made in‐house and used to label transduced cells.

An identical protocol was followed for both. In short, at 1 day in vitro (DIV), 3–5 μL of viral vectors were added to the top of each slice. Experiments were conducted at three different time points: between 8 and 14 DIV, 21 DIV and between 41 and 42 DIV. Infected cells were identified by their green fluorescence (GFP or GCaMP6f).

### 2.3 Calcium imaging and analysis

Occipital cortical slices were transduced with the genetically encoded calcium indicator GCaMP6f under the human synapsin promoter for calcium imaging from neurons. To map evoked postsynaptic activity, slices were imaged at two different time points: between 8 and 13 DIV, denoted as ‘short’ duration cultures, and between 41 and 42 DIV, referred to as ‘long’ cultures. To evaluate spontaneous activity in SP neurons, Ca^2+^ imaging was performed at 14 DIV. All slices were transferred immediately into the submerged recording chamber of an upright VT‐QLC100 spinning disc confocal microscope (Visitech) with PatchStar motorised micromanipulator (Scientifica) and continually perfused (at ~1 to 3 mL/min) with oxygenated artificial cerebral spinal fluid (aCSF) containing (in mM): 126 NaCl, 3 KCl, 1.25 NaH_2_PO_4_, 24 NaHCO_3_, 10 Glucose, 1 CaCl_2_ and 1 MgSO_4_, at approximately 36°C. The orientation of each slice was first identified using a UMPlanFl 10× water objective (NA = 0.3). Tissue was then illuminated with a 491 nm laser (Cobolt Calypso 50) to visualise GCaMP6f positive cells using a LUMPlanFl 40×, 0.8NA objective, and spontaneous calcium transients were captured using a Hamamatsu C9100 EM camera (Hamamatsu Photonics) and VoxCell software (Digital Pixel) at 10 frames per second (fps), for 20–30 s.

In the same brain slices, we also examined Ca^2+^ transients evoked by delivering L‐glutamic acid (1 mm in aCSF; Tocris Bioscience, UK) from a glass micropipette triggered by a short burst of 5 pressure pulses (10 ms pulses, delivered at 10 Hz) generated by a Picospritzer II (Parker Instruments USA). The pipette tip was placed fractionally above the surface of the slice, so that it could be moved across the slice without traumatising the tissue, to assay glutamatergic responses from different sites. Videos of calcium responses were acquired initially at the CP, and the pipette was then moved orthogonal to the pia, in 100 μm steps, to sample responses sequentially through the SP, intermediate/outer subventricular zone (IZ/oSVZ), and inner subventricular/ventricular zone (iSVZ/VZ). Each region was identified based on the cell density, which is low in the SP compared to the neighbouring CP and the subventricular zone. Additional videos were acquired in each slice at the location of the maximal calcium response (typically in the SP region) to examine GABAergic effects. Specifically, we substituted the glutamatergic solution in the delivery pipette with the following solutions: (1) 1 mm GABA (in aCSF, Tocris Bioscience), (2) 0.5 mm glutamate in aCSF, (3) 0.5 mm glutamate/0.5 mm GABA combined in aCSF, (4) aCSF (a control, to test whether the Ca^2+^ response was triggered by the pressure pulse). In each case, the picospritz delivery and the imaging of any Ca^2+^ responses was performed in the identical way to the glutamate stimuli.

In the SP, we additionally examined spontaneous Ca^2+^ activity in the presence of the following bath applied pharmacological agents: gap junction blockers (carbenoxolone, 100 μm), hemichannel blockers (probenecid, 1 mm), GABAA receptor antagonists (gabazine, 10 μm), GABAB receptor antagonists (CGP‐55845, 5 μm), AMPA/kainate receptor antagonists (NBQX/CNQX, 10 μm), NMDA receptor antagonists (APV, 50 μm), and voltage‐gated sodium channel blockers (TTX, 1 μm). After each drug application, slices were washed with aCSF, and a recovery recording was obtained to confirm washout and reversibility of drug effects.

All data processing was conducted using custom‐written code in MATLAB R2024a (MathWorks, RRID:SCR_001622) and FIJI (NIH, RRID:SCR_002285). For evoked postsynaptic activity, a ΔF/F filter with the appropriate threshold was applied to identify cell bodies in each video. The calcium responses were derived from all cell body regions of interest (ROI), and additionally the neuropil response was measured from a region devoid of any cell bodies. The average of the peak fluorescence of each cell body identified, the peak fluorescence of the neuropil, and the number of cells in each frame were also measured for each video acquired. For spontaneous activity, image timeseries were motion corrected using a 2D cross‐correlation approach (Guizar‐Sicairos et al., 2008). Cellular ROIs were drawn using a custom selection tool in FIJI (Cell Magic Wand) (Wilson et al., 2017). Neuropil ROIs were created by drawing an annulus around each cell ROI with a radius of ×4 the cell ROIs, and their signal contamination was removed using a weighted correction factor as previously shown (Dipoppa et al., 2018). ΔF/F0 values were computed using a 30s median filter and a 0.01 Hz low‐pass Butterworth filter to define F0. Activity event detection was done by using the Findpeaks function in MATLAB with the minimum threshold set to 3 standard deviations of the whole trace. The frequency and amplitude of calcium events were calculated for each cell and averaged out for all cells in each time series and slice. All code related to this analysis can be found here: https://github.com/GrimmSnark/paperCodeMcLeod2026_2.

### 2.4 RNA extraction, NanoString transcriptomics and analysis

All cortical slices were snap frozen in liquid nitrogen and stored at −80°C prior to RNA extraction. Purified RNA was extracted from whole slices using the RNeasy Micro kit (Qiagen). In brief, the defrosted slices were homogenised in buffer RLT (lysis buffer) containing β‐mercaptoethanol using a saline syringe (pipetting multiple times), vigorous vortex mixing, and spinning down through a unique biopolymer shredding system (QIAshredder; Qiagen). Subsequently, samples were spun through a series of high‐salt buffers using a microcentrifuge into a silica‐gel membrane which captures the purified RNA. The amount of RNA was then quantified using a Nanodrop Spectrophotometer (Thermo Fisher) and the RNA quality checked with a TapeStation system (Agilent).

The nCounter Pro Analysis System (NanoString) was used to automatically detect gene expression changes over 2 weeks in culture, using their pre‐designed Human Neuropathology gene expression panel (https://brukerspatialbiology.com, XT_Hs_NeuroPath_CSO) supplemented with an additional 24 genes related to neurodevelopment and synaptic function (*ACTB*, *CACNA1*, *CASK*, *CHD2*, *CLTC*, *EFHC1*, *ERBB4*, *GABBR2*, *GPHN*, *GRM3*, *KCNC2*, *NLGN1*, *NRXN2*, *RPLP0*, *SCN9A*, *SLC17A7*, *SLC6A1*, *SNAP25*, *SST*, *STXBP1*, *TRIO*, *TUBB*, *VAMP2*, *WNT7B*), for a total of 794 genes. Approximately 7 μL of RNA (140 ng) was loaded per sample which went through an overnight hybridization step before being placed on the nCounter Pro for a further 12–24 h. The data files (RLF and RCC files) containing the raw counts were then imported into the nSolver Analysis Software 4.0 (NanoString, RRID:SCR_003420) and a series of quality checks were performed including the limit of detection (under 20 counts), presence of contaminants and appropriate binding. Next, background measurements (from blank lanes and internal negative control probe counts) were subtracted from the raw counts. The counts were then normalised to three housekeeping genes (ACTB, MAP2 and TUBB) to account for variations in loading. Finally, the 14 DIV samples were normalised to control samples to calculate the change relative to control values (expressed as a log_2_ “fold‐change”).

Differentially expressed genes (DEG) were assessed with a false discovery ratio analysis and a two‐sided unpaired *t*‐test using R software (RRID:SCR_001905). DEGs were uploaded into Ingenuity pathway analysis (IPA) using the IPA system (Qiagen, RRID:SCR_008653) for subsequent bioinformatics analysis and pathway enrichment, which included canonical pathway analysis and diseases and functions. The *p*‐values were adjusted using the Benjamini–Hochberg (BH) correction to control the false discovery rate (Benjamini & Hochberg, 1995). A log_2_‐fold change of <1 or >1 and a significance level *p* < 0.01 were set to determine which genes were considered differentially expressed. The background gene set was set to all 794 genes in the NanoString panel to ensure calculation of accurate *p*‐values and odds ratios.

All data were mapped to the Ingenuity Knowledge Base to generate an analysis‐ready molecule set. For each pathway or function, IPA calculated the probability that the overlap between the input gene list and molecules annotated to that pathway/function occurred by chance using a right‐tailed Fisher's exact test (equivalent to a hypergeometric test) (Calvano et al., 2005; Cirillo et al., 2017). For display purposes, *p*‐values were plotted on a log_10_ scale. For each significantly enriched term, IPA reported the “top molecules”, defined as the subset of uploaded genes that directly overlap with that biological function. Finally, threshold levels of *p* < 0.01 (−log(*p*‐value) > 2, when plotted as a log scale in the figures) and a ±1 log_2_(fold‐change) (viz. a halving or doubling of expression) were considered noteworthy. The computation of an activation Z‐score to predict activation or inhibition of biological function was based on concordance with curated directional knowledge (Kramer et al., 2014). All Principal Component Analysis (PCA) plots, volcano plots and heat maps were made using custom‐designed code in R software (RRID:SCR_001905).

Analyses were also made of the functional clustering of genes provided by nCounter for the Human Neuropathology panel, to determine if any of these designated subsets of genes were over‐represented within the set of up‐ or down‐regulated genes. For each subset of genes, the baseline probability was set as the number of genes in that subset divided by the total gene number in the Neuropathology panel (760 genes; note we excluded the supplementary 24 genes from these analyses). For example, there were 306 genes within the grouping “Disease associated”, giving a background probability of 0.403 (*p* = 306/760). Using the binomial theorem, we then calculated the probability distributions for each subset of genes for samples of 60 or 71 genes (the number of genes that were up‐ and down‐regulated respectively within the total Neuropathology panel; note that Figure 3 shows larger numbers because these include a handful of genes in the supplementary set of 24 genes which were not included in this analysis). The cumulative probability distributions (binocdf.m run in MATLAB, RRID:SCR_001622) were used to test whether there was an over‐ or under‐representation of genes in a given subset within either the up‐ or down‐regulated set of genes.

Comparisons were made with a previously published data set of acutely processed (non‐cultured) tissue RNAseq data (https://www.hdbr.org/expression; (Lindsay et al., 2016)), from 23 samples of the equivalent brain area (occipital cortex) ranging from 7.5 to 17 pcw. Both data sets were standardized to the combined average expression of ACTB and TBP. For each gene, we used a Monte Carlo simulation to estimate the probability distribution of expected fold‐change, based upon the developmental trajectories. In short, we derived estimates of the predicted mean and standard deviation at 17 and 19 pcw from a linear fit of the acute RNAseq data for each gene. This was then used to simulate a normally distributed set of 1 million paired samples at these two ages, which was in turn used to create the distribution of the expected log_2_(fold‐change). We could then estimate the likelihood of the fold‐change seen in the NanoString data between 0 and 14 DIV (equivalent to 17 to 19 pcw). This was converted to a false‐discovery rate, again using the Benjamini–Hochberg (BH) correction (Benjamini & Hochberg, 1995) (MATLAB, mafdr.m).

### 2.5 Immunohistochemistry, image acquisition and analysis

An immunohistochemistry protocol optimized for thick slices was followed (McLeod et al., 2017). In brief, slices were fixed in 4% paraformaldehyde (PFA)/4% sucrose in phosphate buffered saline (PBS; 0.12 M, pH 7.4) at 4°C overnight and then washed in PBS and incubated in blocking solution (10% donkey serum, 1% Triton X‐100 in PBS) for 4–6 h at room temperature (RT) on a vibrating shaker. Primary antibodies (Table 2) diluted in blocking solution were applied overnight to slices at 4°C on the same vibrating shaker. Slices were then incubated with fluorophore‐bound secondary antibodies (1:500; Life Technologies) for 2–3 h at RT followed by staining with Hoechst (1:5000) and then mounted on glass slides in Fluoromount‐G (SouthernBiotech).

**TABLE 2:** Primary antibodies used for immunohistochemistry.

Primary antibody to	Species	Dilution	Supplier	RRID number
HOMER1	Chicken polyclonal	1:1000	Synaptic Systems	AB_2631222
NR1	Mouse monoclonal	1:2000	Sigma‐Aldrich	AB_ 94,946
GluA2	Rabbit polyclonal	1:1000	Synaptic Systems	AB_2113732
MAP2	Chicken polyclonal	1:1000	Abcam	AB_2138153
GFAP	Chicken polyclonal	1:1000	Abcam	AB_304558
HOPX	Rabbit polyclonal	1:10,000	Thermo Fisher Scientific	AB_2642516
SOX2	Mouse monoclonal	1:500	Santa Cruz Biotechnology	AB_10842165
STXBP1	Rabbit monoclonal	1:500	Abcam	AB_10861492
GFP	Rabbit polyclonal	1:1000	Abcam	AB_303395

Image acquisition of overall slice anatomy, neuronal subtypes, and postsynaptic puncta across cortical laminae was performed using an inverted Zeiss LSM800 Airyscan confocal microscope (Zeiss). Cortical laminae were identified using a 10× objective (NA = 0.45) based on differences in cell density (high in the CP and subventricular zone, and low in the SP), after which tile‐scan z‐stacks spanning the entire slice were acquired with the same objective at 0.5× zoom (z‐step = 5 μm; 2867 × 3789 pixels). Neuronal morphology was analysed from z‐stacks of GFP‐positive neurons acquired from cortical laminae (CP, SP, IZ/oSVZ, and iSVZ/VZ) using a 40× objective (NA = 1.3; 0.5× zoom; z‐step = 5 μm), while postsynaptic puncta were imaged using a 63× objective (NA = 1.4; z‐step = 0.25 μm), with both modalities acquired at a resolution of 2048 × 2048 pixels. Consistent acquisition parameters (e.g. laser power and master gain) were maintained within each imaging set.

Image analysis was performed using custom‐written macros in Fiji (NIH, RRID:SCR_002285; (Schneider et al., 2012)). Image stacks of HOMER1, NR1, and GluA2 were flattened using maximum‐intensity projections, split into individual channels, and the channel of interest was selected. Images were median filtered, thresholded using manually optimised pre‐set values (kept constant across biological replicates for each channel), and segmented using a watershed algorithm to define ROIs. Puncta number and integrated density were quantified per ROI. All measurements were obtained from the raw images. Cell number was determined using Hoechst‐stained nuclei as ROIs, and puncta counts were normalised to nuclei counts to yield puncta count/cell count values.

### 2.6 Statistical analysis

Statistical analyses were performed on GraphPad Prism 10 (RRID:SCR_002798). Data normality was assessed using Kolmogorov–Smirnov tests. Parametric tests were used for normally distributed data, including two‐tailed unpaired *t*‐tests and one‐way ANOVA with Tukey's multiple comparisons test. Non‐parametric tests were used for non‐normally distributed data, including the two‐tailed Mann–Whitney test or Kruskal‐Wallis with Dunn's multiple comparisons test. Correlation between NR1 or GluA2 and HOMER1 puncta count/cell was assessed using simple linear regression, and the relative variance contribution of each protein and of the time in vitro was determined using multiple linear regression. Comparisons were considered to be statistically significant if *p* < 0.05. Data is presented as mean ± SEM. Further details of exact sample replicates and statistical tests used are stated within the Results section and each figure legend.

Multiple organotypic slices were prepared from each human donor and analysed as independent experimental replicates, since each brain slice was incubated in isolation. Slice‐to‐slice variability reflects both biological heterogeneity within the tissue (e.g. spatial origin) and technical variation, and analysing multiple slices improves estimation of within‐donor variability and statistical precision. Given the limited availability of human foetal tissue and associated ethical constraints, this approach is necessary and standard practice.

## 3 RESULTS

### 3.1 Maintenance of neuronal and progenitor cells across cortical laminae in human organotypic slice cultures

We previously established that human developing cortical organotypic slices can be maintained in culture for many weeks, displaying maturation of neuronal networks, while preserving gross laminar organisation (McLeod et al., 2023). After 2 weeks in vitro, distinct cortical wall laminae, including cortical plate (CP), subplate (SP), and the proliferative subventricular zone (SVZ), remained readily identifiable (Figure 1). Immunofluorescence analysis revealed the sustained presence of differentiated neurons and multiple progenitor populations across these laminae. MAP2‐ and TBR1‐positive post‐mitotic neurons were enriched within the CP and SP, while progenitor markers identified heterogeneous radial glial populations within proliferative zones, including GFAP‐positive radial glia, HOPX‐positive basal radial glia, and SOX2‐positive apical radial glia. Ubiquitous labelling with AAV9‐CMV‐GFP further demonstrated extensive morphological diversity, labelling neurons and radial glial subtypes across laminae, including ventricular and outer radial glial cells (Figure 1).

**FIGURE 1:**
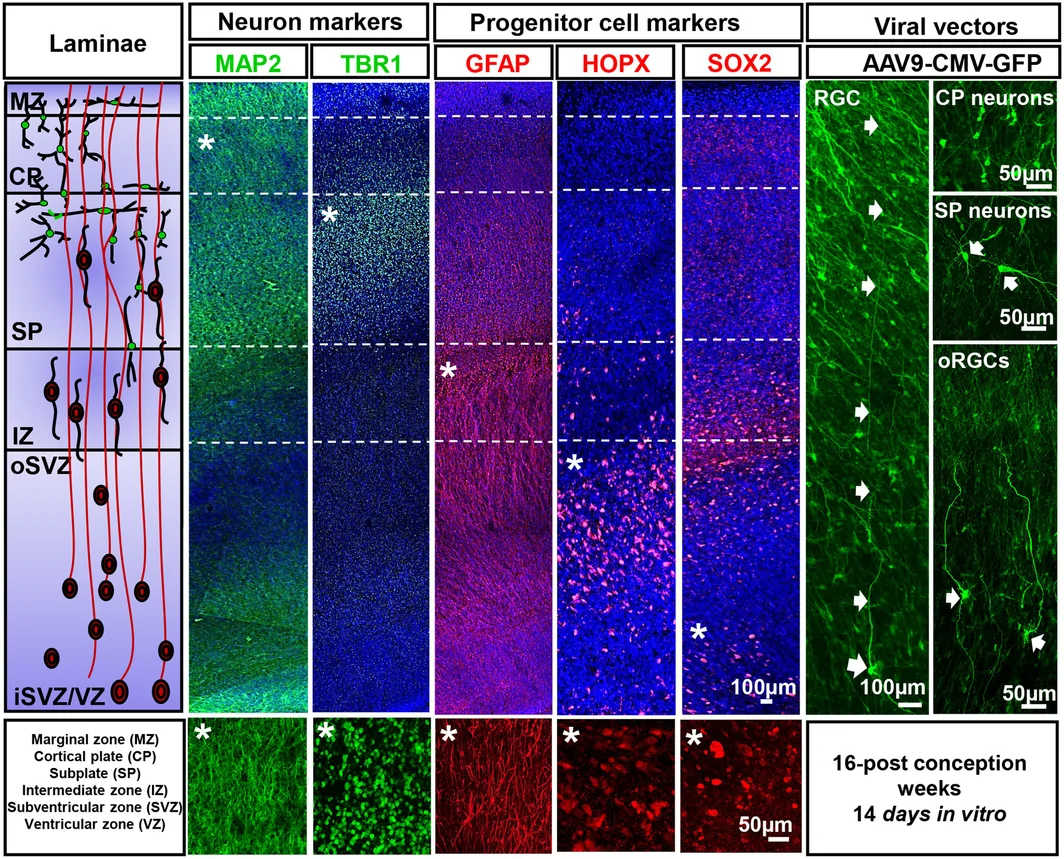
Sustained maintenance of diverse neuronal and progenitor populations in vitro. Left panel: Schematic representation of the distinct cortical laminae, including the marginal zone (MZ), cortical plate (CP), subplate (SP), intermediate zone (IZ), outer/inner subventricular zone (o/iSVZ), and ventricular zone (VZ). Middle panels (top): Representative immunofluorescence images showing mature neuronal markers (MAP2 and TBR1) and progenitor cell markers, including the pan–radial glial marker GFAP, the basal radial glial marker HOPX, and the apical radial glial marker SOX2. Middle panels (bottom): Higher‐magnification views (starred in top panel) of the corresponding regions for each marker. Right panel: Representative images showing the diverse cell types labelled by the viral vector AAV9‐CMV‐GFP, which broadly labels all cells, including CP neurons, SP neurons, outer radial glial cells (oRGCs), and long‐range radial glial cells (RGCs). White arrows highlight axons and cell bodies. All images taken from a 16‐post conception week sample (pcw), 14 days in vitro.

To visualise neuronal morphology and distribution, neurons were transduced with the GFP‐based reporter GCaMP6f driven by the Synapsin‐1 promoter. At 14 DIV, most GFP labelled neurons were located in the SP, where we also observed punctate immunoreactivity for STXBP1, a pre‐synaptic protein. GFP labelled neurons and STXBP1 immunoreactivity were also observed in the intermediate zone (IZ) and outer subventricular zone (oSVZ) but were absent from the CP (Figure 2(a)). Small clusters of cells near the basal surface of the CP may represent remnants of the marginal zone, a thin layer which nevertheless resembles the SP in terms of neuronal maturation and synapse formation in vivo (Bayatti et al., 2008; Kostovic et al., 2019; Kostovic & Rakic, 1990) but does not seem to survive well ex vivo.

**FIGURE 2:**
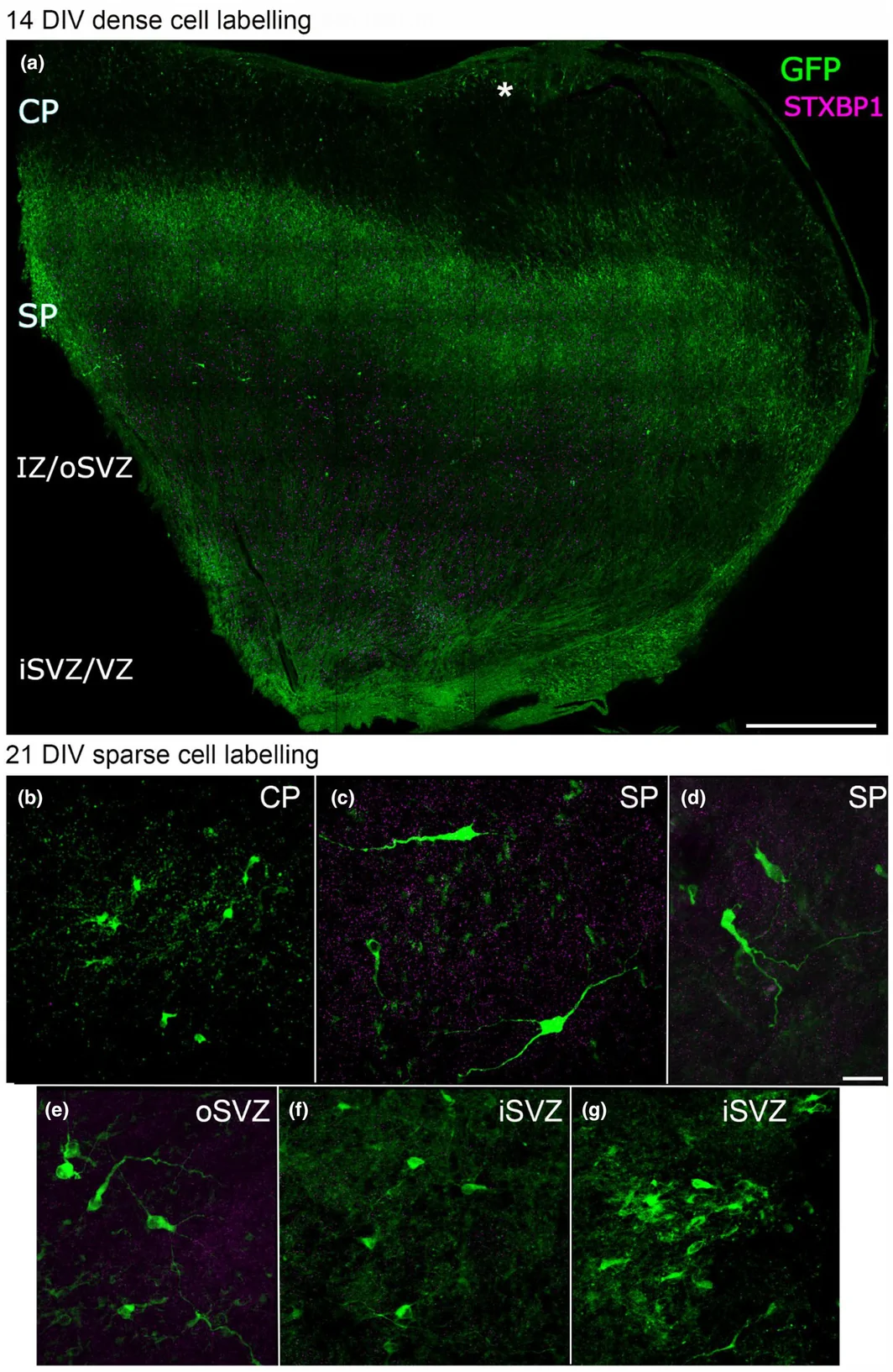
Morphological characterisation of GCaMP6f‐positive neurons in different cortical laminae. In all figures the basal (pial) pole of the slice is towards the top and the apical (ventricular) pole towards the bottom. (a) Low power view of an entire 16 pcw slice after 14 DIV with a high level of transduction with GCaMP6f. GFP (green) labelled neurons localised predominantly to the subplate (SP) with only sparse representation in the cortical plate (CP) although some cells can be seen near the pial surface (asterisk) that might be a remnant of the marginal zone. Some GFP‐positive neurons are also present in the intermediate zone (IZ) outer subventricular zone (oSVZ) and inner subventricular zone and ventricular zone (iSVZ/VZ). STXBP1 immunofluorescence (magenta) appeared punctate and was most prominent in the SP, IZ and oSVZ. (b–g) High power images of GFP‐positive neurons after 21 DIV—small CP neurons (b), typical larger tangential bitufted SP neurons alongside a small neuron (c), two monotufted SP neurons (d), smaller neurons in the oSVZ (e) and examples of single and clustered small GFP‐positive neurons in the iSVZ (f, g). Note STXBP1 immunoreactivity in SP and oSVZ but not the CP and iSVZ. Scale bars in a = 1 mm; d also for b, c, f, g = 50 μm.

At 21 DIV, neurons in the CP appeared small and quite rounded with multipolar processes which were thin and difficult to trace (Figure 2(b)). In the SP, GFP+ neurons were more numerous and generally quite large. The morphologies we observed have been previously described for SP neurons following biocytin filling in rat (Hanganu et al., 2002). These included bitufted neurons with dendrites extending over several hundred microns horizontally across the cortical wall (Figure 2(c)). These processes were thicker than those arising from CP neurons. Another common type resembled the horizontal monotufted neuron with a large primary dendrite splitting into two or more dendritic processes (Figure 2(d)) that could extend radially or horizontally. We saw fewer large multipolar neurons in our cultures than have been reported in rat (Hanganu et al., 2002) or in human Golgi preparations (Kostovic et al., 2019).

Smaller neurons were also observed, such as in Figure 2(c), with a single neurite extending up towards the CP, which may be a radially migrating neuron. oSVZ neurons were generally smaller than SP neurons, and these showed a variety of morphologies and orientations. Closer to the apical pole of the culture, there were clusters of GFP+ cells as well as isolated ones (Figure 2(f)–(g)). The expression of GFP suggests that these were comparatively more mature neurons than the majority of neurons found in the CP. Despite the synapsin promoter being active, there was no immunoreactivity for pre‐synaptic STXBP1 close to these cells, unlike in the SP and oSVZ (Figure 2(c)–(e)).

### 3.2 Global transcriptomic remodelling over time in culture

To explore the developmental progression within the cultures, we performed transcriptomic profiling; this provides an unbiased, systems‐level readout of gene expression and can help identify coordinated changes of expression. We prepared 26 occipital cortical slices from three human donors at 17 pcw; 8 slices were frozen immediately after preparation (0 days in vitro, DIV), while 18 slices were maintained for 14 days in vitro (14 DIV). before processing all the slices together for RNA extraction and analysing 794 target genes using the NanoString platform. The 1st principal component, accounting for 62.0% of the variance, revealed a clear separation between slices from the 0 and 14 DIV samples, indicating that these cultures display an evolving transcriptomic profile (Figure 3(a)). Interestingly, the 2nd principal component (10.1% of the variance) also separated slices from one brain from those derived from the other two brains at 14 DIV, although this separation was not observed at 0 DIV.

**FIGURE 3:**
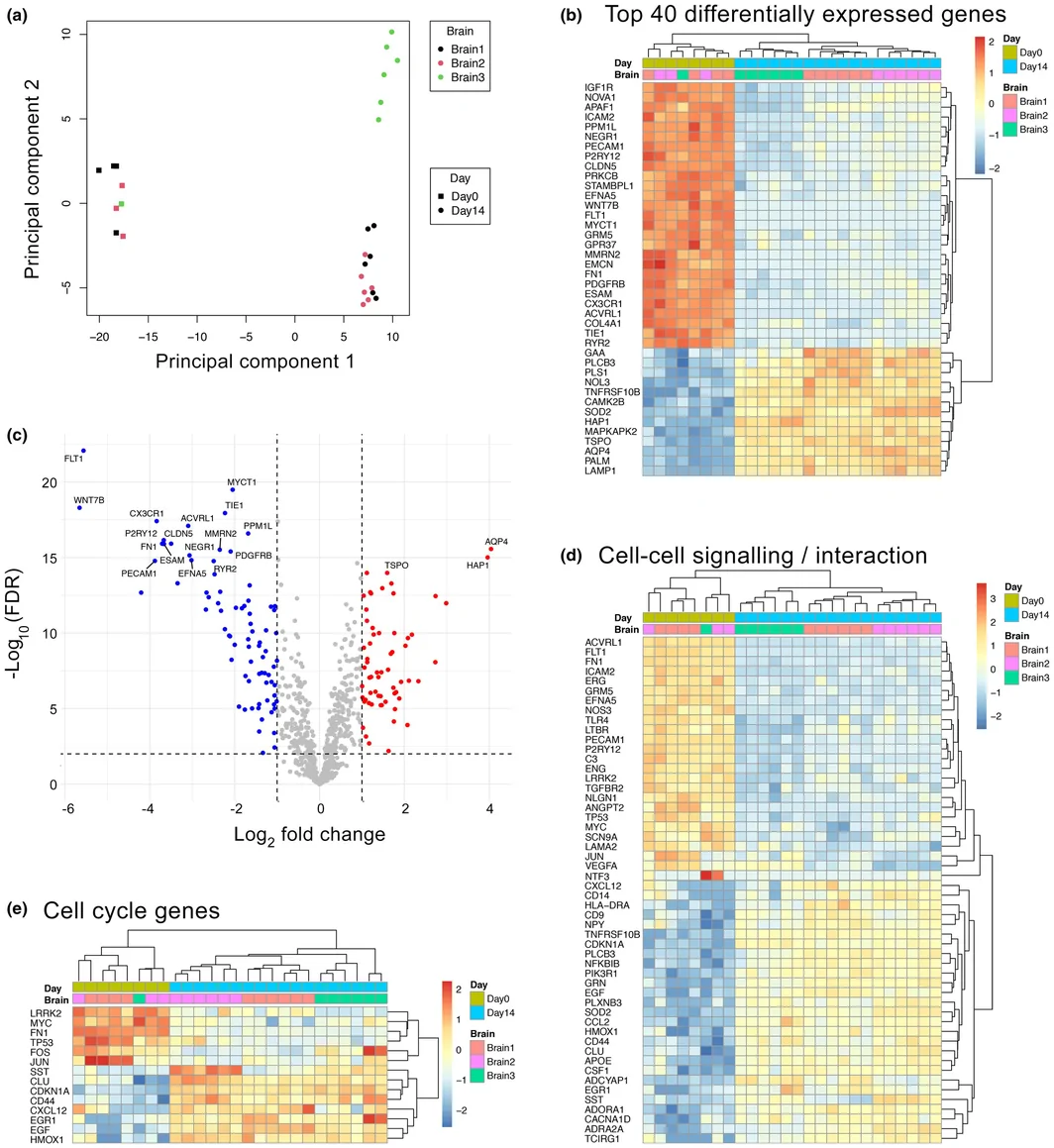
Dynamic transcriptomic reorganisation after 2 weeks in culture (a) Principal component analysis (PCA) of the gene expression across all samples prepared from 3 different brains, at 0 and 14 DIV. The 1st principal component, which separates the 0 and 14 DIV data, represents 62.0% of the variation, and the 2nd, which separates brain 3 from the other two samples, represents just 10.1%. (b) Volcano plot displaying the upregulated (red) and downregulated (blue) genes at 14 DIV (relative to 0 DIV) with two‐sided unpaired *t*‐test. Differentially expressed genes (DEG) were defined as those with more that 1‐fold change and *p* < 0.01; unchanged genes are shown in grey. (c) A heatmap showing the top 40 differentially regulated genes (both up‐ and down‐regulated) for the 18 samples. Cladograms at the top and right show the clustering patterns of the samples and the genes respectively. Similarly arranged heatmaps for the top two pathways identified by the Ingenuity Pathway Analysis: “cell‐to‐cell signalling and interaction” (d) and “cell‐cycle” (e) pathways. Note that each sample cladogram separates the 3 brains at 14 DIV, but observe also how consistent the changes are across the samples between 0 and 14 DIV.

Differential expression analysis identified a total of 134 (16.9%) genes that changed at the designated threshold levels (log_2_(fold change) exceeds ±1, and the false‐discovery rate (adjusted *p* value) < 0.01) between 0 and 14 DIV, with 62 being raised at 14 DIV, and 72 being reduced (Figure 3(b), (c)). The top ten up‐regulated genes are listed in Table 3, and the top ten down‐regulated ones in Table 4. To identify coordinated patterns of gene expression change between 0 and 14 DIV, the differentially expressed genes were subjected to Ingenuity Pathway Analysis (see Methods). Of the biological functions examined in these analyses, the two most prominent were the “Cell‐to‐cell signalling and interaction” and the “Cell cycle” pathways (Figure 3(d), (e)). Again, note that while the cladogram does still separate the three brains for both pathway analyses, overall, the heatmap visualisation of genes contributing to these biological functions revealed relatively consistent expression changes across all the samples between 0 and 14 DIV. Across all transcripts, there was marginally greater heterogeneity of expression in 14 DIV slices (see also the PCA, Figure 3(a); for all transcripts, coefficient of variation (CV) for 0 DIV = 0.1944 ± 0.0055; 14 DIV CV = 0.2111 ± 0.0048; *p* = 0.0023, paired Student's *t*‐test).

**TABLE 3:** Top 10 up‐regulated genes in 14 DIV cultures. The asterisked genes are designated as “Disease Associated” (nCounter).

Gene	Log_2_ fold‐change	Gene product and functions	References
*AQP4* ^a^	4.04	Aquaporin 4; astrocyte marker with role in stress response	Li et al. (2011)
HAP1*	3.96	Hungtintin‐associated protein 1; promotes organelle transport, synaptic plasticity, and GABA receptor recycling	Zhao et al. (2022)
*CDKN1A* ^b^ *	2.99	p21 protein; Upregulated by p53. Cell stress response. Inhibits cell division, promotes DNA repair/apoptosis.	Marques‐Torrejon et al. (2013)
*TNFRSF10B* ^b^ *	2.73	Death receptor (DR) 5; Upregulated by p53. Promotes apoptosis in response to DNA damage and ER stress.	Lam et al. (2020)
*CCL2* ^a^ *	2.73	Chemokine; Recruits and activates microglia	Cherry et al. (2020)
*HLA‐DRA* ^a^ *	2.33	Class II major histocompatibility antigen; Activated microglia marker.	Mattiace et al. (1990)
*CNTF* ^c^ *	2.18	Ciliary neurotrophic factor; Astrocyte marker. Neuroprotective, promotes neurotransmitter synthesis and neurite outgrowth.	Guo et al. (2022)
*APOE* ^a^ *	2.11	Apolipoprotein E; Astrocyte and activated microglia marker.	Strickland et al. (2026)
*NQO1* ^c^	2.08	Neuroprotective; protects cells against oxidative stress	Kim et al. (2013)
*EGR1* ^b^ *	2.07	Transcription factor; induced by growth factors and cellular stress. Upregulates p53 expression.	Krones‐Herzig et al. (2003)

^a^ Associated with astrocytic or microglial activation in response to cell stress or neuroinflammation.

^b^ Associated with the p53 regulated tumour suppressor pathway.

^c^ Neuroprotective molecules.

**TABLE 4:** Top 10 down‐regulated genes in 14 DIV cultures.

Gene	Log_2_ fold‐change	Gene product and functions	References
*WNT7B*	−5.65	Secreted protein; ligand for Wnt/β‐catenin signalling. Involved in tissue morphogenesis, synapse formation.	Ferrari et al. (2018)
*FLT1* ^*a*^	−5.56	VEGF receptor 1; key role in inhibiting angiogenesis during development	Fischer et al. (2008)
*GRM5*	−4.20	Metabotropic glutamate receptor 5; post‐synaptic glutamate receptor, highly expressed in development	Romano et al. (1996)
*PECAM1* ^*a*^	−3.88	CD31; expressed by endothelial and immune cells, maintains endothelial tight junctions; mechano‐sensation, inhibits transmigration of leukocytes	Woodfin et al. (2007)
*CX3CR1* ^a^, ^b^	−3.84	Cell surface receptor expressed by microglia and endothelial cells. Promotes cell migration, angiogenesis and apoptosis resistance	Clark et al. (2011)
*FN1*	−3.71	Fibronectin‐1; promotes cell migration including cortical neuron interaction with radial fibres	Peregrina and Del Toro (2020)
*P2RY12* ^b^	−3.67	Purinergic receptor; marker for resting microglia, reduced expression after microglial activation.	Haynes et al. (2006)
*ESAM* ^a^	−3.67	Endothelial cell adhesion molecule; maintains integrity of blood vessels/blood brain barrier in development	Lecca et al. (2023)
*CLDN5* ^a^	−3.50	Claudin‐5; maintains integrity of blood vessels/blood brain barrier	Hashimoto et al. (2023)
*PRKCB*	−3.34	PKC‐beta; PKC signalling promotes cell survival/inhibits apoptosis	Antal et al. (2015)

^a^ Associated with the integrity of blood vessels and the blood brain barrier.

^b^ Marker for quiescent microglia.

We examined whether different functionally defined sets of genes were over‐ or under‐represented within identified sets of up‐ and down‐regulated genes. Across the 24 different “Annotation” groups, and 6 different “Theme” groups provided by nCounter (https://brukerspatialbiology.com), only one showed a distribution pattern that differed from random (Figure 4), namely the “Disease Association” genes, which were over‐represented within the upregulated genes set (36 out of 60 genes; *p* = 0.0016). This over‐representation was also evident within the top 10 genes (Table 3; 8 out of 10 genes; *p* = 0.0018). Notably, none of the other functional groupings were over‐represented in either the up‐ or the down‐regulated group of genes.

**FIGURE 4:**
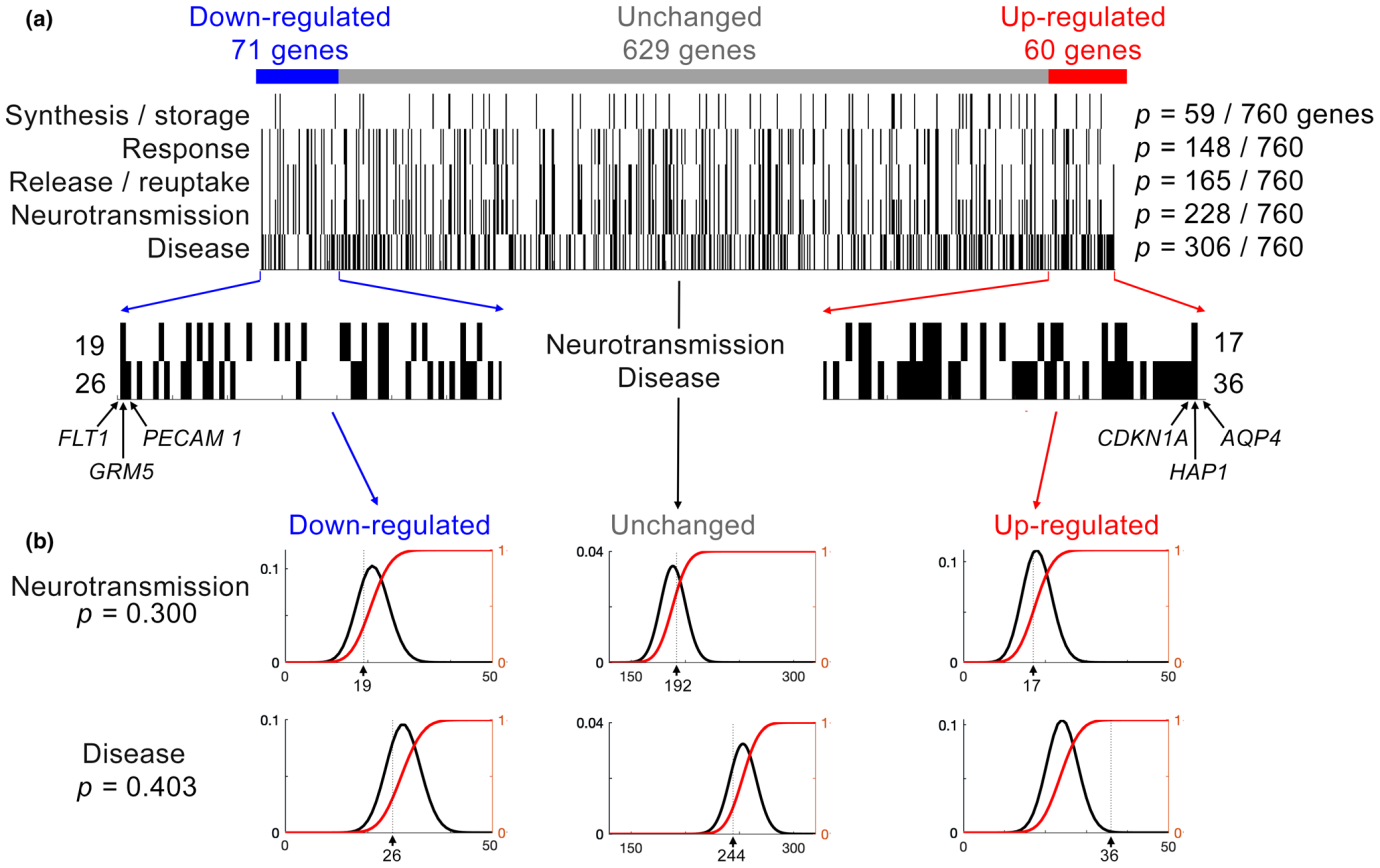
Genes encoding for neurotransmission function are preserved in organotypic cultures. (a) The distribution of genes in 4 of the specified functional groupings (“neurotransmitter synthesis & storage”, “neurotransmitter response”, “neurotransmitter release and reuptake” and “disease associated” (nCounter, https://brukerspatialbiology.com); the first 3 were then combined into the general group “neurotransmission”). The genes are ordered with respect to how expression changes between 0 and 14 DIV. The probability of a gene being in the different groups are listed at the right (e.g. there were 306 genes within the “disease associated” group, out of a total of 760 genes, giving *p* = 0.403; note that the 24 genes in the supplementary group were not included in this analysis). 19 “neurotransmission” and 26 “disease” genes were in the down‐regulated set of genes at 14 DIV, and 17 and 36 genes in these two functional groups respectively were in the up‐regulated gene set, as shown in the enlarged panels. (b) The probability distributions for the numbers of “neurotransmission” and “disease” genes expected to be found in the down‐ and up‐regulated gene sets, and also the “unchanged” set, if those gene groups were randomly distributed across the entire set at the probabilities shown. These are calculated using the binomial theorem, where the mean is n*p*, and the standard deviation is √ (n*p*(1*‐p*)) (e.g. for the top left panel, the mean, n*p* = 0.3 × 71 = 21.3). Note that for 5 out of the 6 distributions show, the actual number of genes is close to the middle of the expected distribution and in each case, non‐significant. Only for the number of “disease” genes in the up‐regulated set (36/60, bottom right panel) is this significantly different from the expected mean of 24.18 (n*p* = 0.403 × 60).

Upregulated gene expression included a group expressed more strongly by astrocytes and microglia in response to stress (e.g. *AQP4*, *APOE*, *CCL2*, *HLA‐DRA*; see Table 3), while the genes that showed downregulated expression included those that are known to be suppressed following microglia activation (*CX3CR1*, *P2RY12*; see Table 3). We conclude that the culturing process does subject the different classes of glia to some degree of cellular stress. Another highly upregulated group includes members of the p53 pathway (e.g. *CDKN1A*, *TNFRS10B*, *EGR1*; see Table 3) which responds to cell stress by halting cell division to allow for DNA repair or alternatively triggering apoptosis (Wang et al., 2023). However, it should be noted that expression of *TP53*, which codes for p53, is slightly downregulated (Figure 3(e)). Neuroprotective factors (*CNTF*, *NQO1*) are also highly upregulated, while a potential inhibitor of apoptosis, *PKRCB*, was significantly downregulated.

Among the other most down‐regulated genes are several involved in angiogenesis and establishment of vascular structural integrity (e.g., *FLT1*, *PECAM1*, *ESAM*, *CLDN5*; Table 4), suggesting that disconnected blood vessels are not maintained. Finally, *FN1*, which codes for fibronectin‐1 a molecule implicated in cell migration along radial glial fibres, was also significantly down‐regulated (Table 4).

The Neuropathology panel includes 228 genes that are associated with neurotransmission (sub‐grouped into “Release”, “Response and Reuptake”, “Storage and synthesis”), but all groups showed an evenly distributed mix of up‐, down‐regulated, and unchanged expression (Figure 4).

These data suggest that there is coordinated gene expression changes between 0 and 14 DIV but leaves open the question to what extent these represent a continuation of the normal developmental pathway, or an adaptive response to the culture methodology. Ideally, we would compare tissue of the same biological age (viz. comparing 17 pcw samples that were then cultured for 14 DIV (p17/14) with 19 pcw/0 DIV tissue (p19/0)), but samples were only available until 18 pcw, so this comparison could not be made. Instead, we used a previously collected set of non‐cultured tissue RNAseq data (https://www.hdbr.org/expression; (Lindsay et al., 2016)) from 23 samples of the equivalent brain area (occipital cortex) ranging from 7.5 to 17 pcw, to estimate the expected p19/0 state by extrapolation of this dataset beyond 17 pcw, the average age of samples used in this study. To align the different datasets, we identified two housekeeping genes, β‐actin (*ACTB*) and TATA‐box binding protein (*TBP*) that showed constant expression across both the developmental series, and also between 0 and 14 DIV. When we normalized the transcript levels for genes to the average expression for *ACTB* and *TBP*, the 0 DIV data at 17 pcw (p17/0) matched the developmental series very well for the most genes examined (Figure 5). We were then able to determine, for each individual gene, whether the subsequent p17/14 data followed the in utero developmental trajectories (extrapolated to p19/0) or diverged from these. Across all the genes in our NanoString panel, 166 (20.9%) showed significant correlations with the developmental age, indicative that their gene expression was systematically increasing (105 genes, 13.2%) or decreasing (61 genes, 7.7%). Comparing these trajectories with the gene expression changes following 14 DIV in culture, we found good matches in the patterns of change (35 of 62 upregulated genes (56%) were showing upwards trajectories from 7.5 to 17 pcw; 518 of 653 unchanged genes (79%) also showed no correlation of in utero age with expression level; 38 of 78 downregulated genes (49%) were following a downwards trajectory).

**FIGURE 5:**
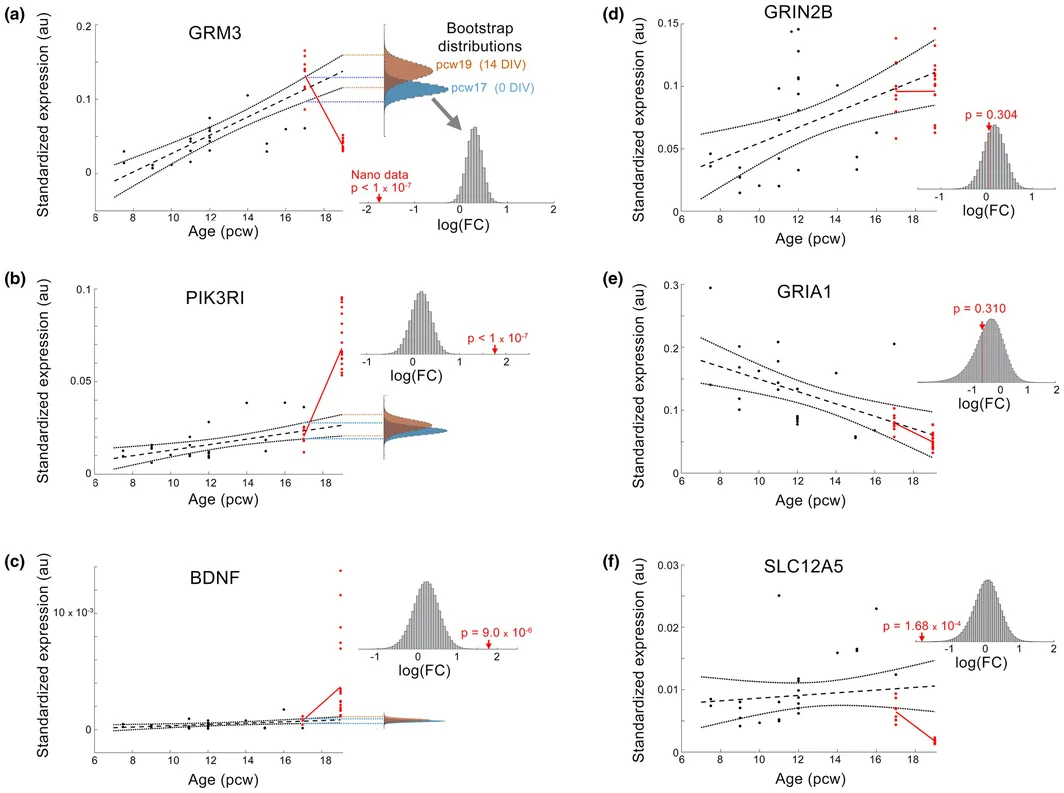
Comparisons of gene expression changes in culture with normal developmental trajectory. In utero, developmental changes in expression (black, RNAseq data), together with the changes seen in organotypic cultures (red, NanoString data), are shown for 6 different genes to illustrate the variety of patterns seen. For each plot, the standardized data from acutely prepared fresh samples (black) at the different post‐conceptual weeks (pcw), together with the NanoString data (red) from the organotypic cultures at 0 DIV (equivalent to 17 pcw) and 14 DIV (equivalent to 19 pcw). A linear regression (black dashed line) is fitted to the acutely processed samples (the black data and the 0 DIV samples), and the curved lines represent the 95% confidence intervals. For each gene, we used a bootstrap approach to simulate the distribution of fold‐change from 17 to 19 pcw. This was accomplished first by simulating distributions of 1 million paired samples at these two time points, based on the fitted estimates of the mean and standard deviation (blue, 17 pcw; brown, 19 pcw), and then calculating the log_2_(fold‐change) for each pair (grey histograms). The red arrow indicates the actual log_2_(fold‐change) between 0 and 14 DIV, and the probability of such an occurrence, based upon the simulated probability distribution function (pdf). Note that where genes are showing a strong upwards trajectory, the bootstrapped pdf is biased towards positive values (GRM3, PIK3RI, GRIN2B), whereas genes showing a downwards trajectory, the bootstrapped pdf is biased towards negative values (GRIA1). The examples represent the full spectrum of effects, including two with highly significant down‐regulation (GRM3, SLC12A5), two with highly significant up‐regulation (PIK3RI, BDNF), and two where the NanoString data matches very well with the RNAseq data.

To investigate this further, we used Monte Carlo simulation to estimate the probability distribution functions of the fold‐change in expression between 17 and 19 pcw for normal, in utero development (equivalent to the 0–14 DIV NanoString data) using the estimates for the mean and standard deviation at those two time points (Figure 5) for each gene. We did this by creating a random set of 1 million paired expression levels, and from this, derived a simulated distribution of log_2_(fold‐change), thus providing a frame of reference for the actual measured fold‐change from the NanoString data (red arrows in Figure 5). Figure 5 illustrates this process and shows examples of different genes representing the full range of gene‐expression changes. Correcting for the sample size using the Benjamini–Hochberg (BH) correction (Benjamini & Hochberg, 1995), we find that just 48 out of 793 genes (6.1%; *p*, 0.01; note 1 gene in the NanoString data set was not represented in the HDBR set) showed upwards regulation that was significantly higher than that predicted by the extrapolated acute RNAseq dataset, and 83 out of 793 genes (10.5%) showed significant drop. The genes showing significant deviation from the normal developmental trajectory following 2 weeks in culture are listed in Table 5. In other words, for the majority (83.4%) of genes of genes, the measured changes in expression in culture followed developmental trends that were already evident in acute samples of tissue taken between 7.5 and 17 pcw (Figure 5).

**TABLE 5:** In both groups, the genes are listed in order of the largest change.

**Up‐regulated genes:** *HAP1*, *HLA‐DRA*, *CNTF*, *NQO1*, *EGF*, *BDNF*, *PIK3R1*, *PLS1*, *CSF1*, *CA2*, *CAMK2B*, *NEFL*, *CLU*, *MTHFR*, *PLA2G16*, *PSMB9*, *SOD2*, *CPT1B*, *ADRA2A*, *SLA*, *WFS1*, *LAMP1*, *GRN*, *ACAA1*, *ITPR2*, *DDIT3*, *MYD88*, *RAPGEF2*, *ADCY5*, *KCNB1*, *NPC2*, *NFE2L2*, *FGF12*, *BAX*, *ATP6V0D1*, *ACTN1*, *SQSTM1*, *COMT*, *AARS*, *CPLX1*, *HEXB*, *PPP2R5E*, *BECN1*, *PFN1*, *AP2A2*, *AKT1S1*, *C9orf72*, *IDE* **Down‐regulated genes:** *WNT7B*, *FLT1*, *GRM5*, *PECAM1*, *CX3CR1*, *FN1*, *ESAM*, *CLDN5*, *PRKCB*, *ACVRL1*, *NEGR1*, *EFNA5*, *C3*, *GPR37*, *STAMBPL1*, *RYR2*, *MMRN2*, *ICAM2*, *ANGPT2*, *NLGN1*, *PDGFRB*, *PCSK2*, *FGF14*, *NOSTRIN*, *ERBB4*, *SLC12A5*, *GRM3*, *HCN1*, *PPM1L*, *SCN9A*, *PLLP*, *XK*, *OLFM3*, *ADCY8*, *GPR4*, *FRMPD4*, *PLCB4*, *IPCEF1*, *NOS3*, *TLR4*, *PLXNC1*, *TENM2*, *LTBR*, *RPLP0*, *NTNG1*, *APAF1*, *IGF1R*, *NOVA1*, *PLA2G4C*, *GALC*, *RIMS1*, *SP100*, *NRG1*, *GABBR2*, *CRH*, *PPARGC1A*, *CHRNA7*, *SYT13*, *CAMK4*, *EPHA6*, *ATXN3*, *GLRB*, *INSR*, *ATP6V1G2*, *AKT3*, *SIRT1*, *GLS*, *CUL3*, *SPAST*, *CACNB2*, *HDAC2*, *MNAT1*, *NRXN1*, *CHL1*, *SLC1A2*, *GNG2*, *MTO1*, *GPHN*, *PIK3CA*, *DGKE*, *CAB39*, *BACE1*, *ACTB*

### 3.3 Subplate neurons form spontaneously active synaptic networks

The prominent levels of synaptic protein expression in these cultures indicate that neurons are likely to exhibit functional synaptic communication. To examine this directly, we transduced neurons in occipital cortical slices with the genetically encoded calcium indicator GCaMP6f under the Synapsin‐1 promoter (Figure 6(a)) to allow visualisation of neuronal activity across the network. At 14 DIV, neurons showed spontaneous activity in the subplate (Figure 6(b)), which was significantly increased in rate either by blocking GABAergic inhibition using gabazine and CGP‐55845 to block GABA_A_ and GABA_B_ respectively, or blocking gap junctions using carbenoxolone (100 μm) with probenecid (1 mm; control slices = 0.14 ± 0.008 events/s (Hz); GABA block = 0.17 ± 0.006 Hz; gap junctions/hemichannels blockade = 0.21 ± 0.01 Hz; *p* < 0.001, one‐way ANOVA with Tukey's multiple comparisons test, Figure 6(c)). Interestingly, the amplitude of Ca^2+^ transients decreased in the presence of gap‐junction blockers (control = 0.19 ± 0.02 ΔF/F0; gap junctions/hemichannels = 0.09 ± 0.007 ΔF/F0; *p* < 0.001, one‐way ANOVA with Tukey's multiple comparisons test, Figure 6(d)), although there was no change in amplitude with GABAergic blockers (0.23 ± 0.01 ΔF/F0; *p* > 0.05, one‐way ANOVA with Tukey's multiple comparisons test, Figure 6(d)).

**FIGURE 6:**
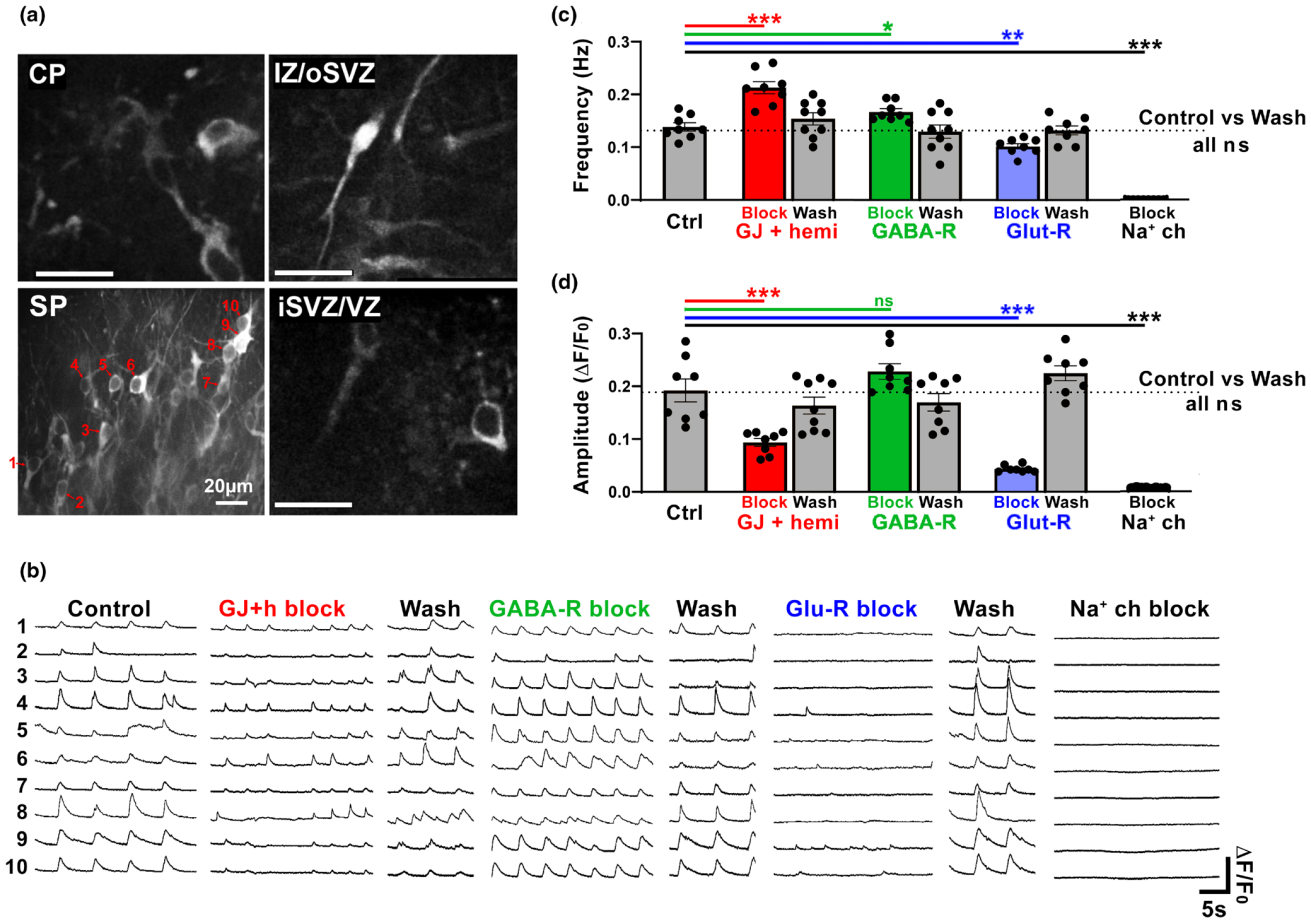
Calcium transients in the subplate are strongly dependent on glutamatergic transmission and action potential firing. (a) Representative frames of the calcium responses visualised with GCaMP6f, from the cortical plate (CP), intermediate/outer subventricular zone (IZ/oSVZ), and inner subventricular/ventricular zone (iSVZ/VZ) and the subplate (SP) in 14 DIV slices prepared from a 16 pcw foetal brain. The numbered cells in the SP micrograph refer to the traces in panel (b), which shows spontaneous neuronal calcium events recorded in the absence or presence of pharmacological blockers of gap junctions/hemichannels (carbenoxolone and probenecid), GABAergic transmission (gabazine and CGP‐55845), glutamatergic transmission (NBQX, CNQX, and APV), or voltage‐gated sodium channels (TTX). All scale bars = 20 μm. (c) Event frequency. Calcium event frequency increased with gap junction/hemichannel blockers (***p < 0.001) and GABAergic blockers (*p < 0.05), decreased with glutamatergic blockers (**p < 0.01), and was nearly abolished by TTX (*p* < 0.001). (d) Event amplitude. Calcium event amplitude was significantly reduced by gap junction/hemichannel blockers, glutamatergic blockers, and TTX (all *p* < 0.001). There was no statistical difference for any of the wash‐out recordings compared to the initial baseline (control) recordings, for either amplitude or frequency measures. All statistical significance assessed by one‐way ANOVA with Tukey's multiple comparisons test. The scatter plots in (c, d) show individual slice means (*n* = 8 slices per condition from three 16–17 pcw human donors) and the histograms show the collective slice mean ± SEM.

In contrast, blocking AMPA and NMDA glutamatergic receptors with a cocktail of NBQX, CNQX (both 10 μm) and APV (50 μm) led to a significant reduction in both rate (0.10 ± 0.005 Hz, *p* < 0.01, one‐way ANOVA with Tukey's multiple comparisons test) and amplitude of events (0.04 ± 0.002 ΔF/F0; *p* < 0.001, one‐way ANOVA with Tukey's multiple comparisons test), and blockade of voltage‐gated Na^+^ channels completely abolished calcium transients (Figure 6(b)–(d)).

### 3.4 Evoked neuronal activity higher in the SP and SVZ than in the CP

We further explored network excitability by mapping evoked post‐synaptic responses to focal, picospritz‐delivery of glutamate (1 mm in aCSF) in the CP, SP, IZ/oSVZ, or iSVZ/VZ at two different durations of culture (‘short’ (8–13 DIV) and ‘long’ (41–42 DIV); Figure 7(a)) prepared from 16 to 18 pcw samples. We found a marked variation in the responsiveness to glutamatergic stimulation between different laminae in the long culture tissue, in terms of the amplitude of the response in cell somata (ΔF/F: CP = 7.85 ± 3.92, SP = 48.6 ± 8.78, IZ/oSVZ = 28.1 ± 8.82, iSVZ/VZ = 53.1 ± 13.8, *p* = 0.01, one‐way ANOVA with Tukey's multiple comparison test; Figure 7(b)) and also the neuropil response (ΔF/F: CP = 1.53 ± 0.437, SP = 13.2 ± 4.21, IZ/oSVZ = 2.21 ± 0.803, iSVZ/VZ = 9.31 ± 3.64, *p* = 0.01, Kruskal‐Wallis with Dunn's multiple comparison test; Figure 7(c)). Averaged across all samples, responses were highest in the SP, followed by those in iSVZ/VZ. In over half the slices, the peak response was located in the SP (9/16 slices, 56%); in 3 other slices, the peak response was in the adjacent IZ/oSVZ (19%), in the iSVZ/VZ in 3 slices (19%), and in the CP in just a single slice (6%). This pattern of maximal responses in the central laminae (SP and IZ) was also evident in the short duration cultures, although the differences between laminae were not significant (somatic ΔF/F: CP = 24.26 ± 7.53, SP = 30.38 ± 1.91, IZ/oSVZ = 27.11 ± 6.37, iSVZ/VZ = 18.89 ± 5.80, *p* = 0.59, Figure 6(c); neuropil ΔF/F: CP = 4.59 ± 1.34, SP = 6.00 ± 0.695, IZ/oSVZ = 4.68 ± 1.68, iSVZ/VZ = 1.71 ± 0.441, *p* = 0.13; both tests one‐way ANOVA with Tukey's multiple comparison test; Figure 6(d)). One point to note is that we consistently found GCaMP6f‐expressing cells deep in the proliferative zones, and these presumptive neurons could be activated by glutamate.

**FIGURE 7:**
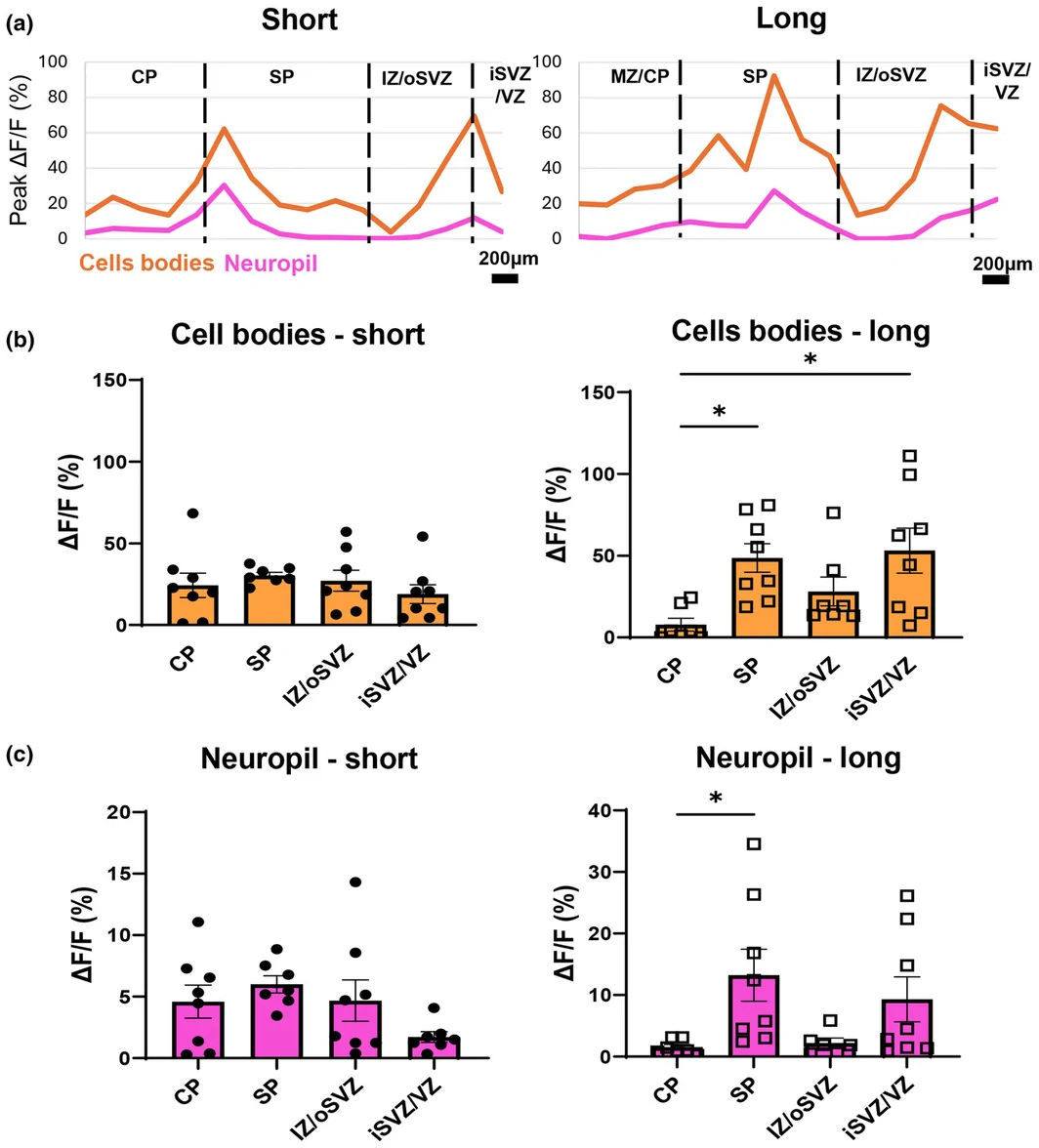
Glutamate evoked calcium response across different regions of human organotypic slice cultures. (a) Representative plots of the peak calcium response in an individual slice to glutamate (1 mm) measured as ΔF/F at the short (8–13 DIV; left) and long (41–42 DIV; right) culture time points. Peak fluorescence values are shown for the cell body (orange) and neuropil (pink). (b) Quantification of the peak calcium response to glutamate per region at the short (left) and long culturing time points (right), measured as the mean fluorescence of each cell detected shows a significant increase in the SP and iSVZ/VZ, compared to the CP, in the long cultures (*p* < 0.05 for both). (c) Quantification of the peak calcium response to glutamate per region at the short (left) and long cultures (right), measured as neuropil showed a significant increase in the SP compared to the CP at the long cultures (*p* < 0.05). All data in (b, c) presented as scatter plots displaying slice data points with mean ± SEM and *n* = 8 slices per DIV group from two (16 and 18 pcw) human donors. Statistical significance assessed by one‐way ANOVA with Tukey's multiple comparisons test or Kruskal‐Wallis with Dunn's multiple comparisons test.

We next tested the effects of GABA delivery at the site of the maximal glutamatergic response in each slice (typically in the SP). During development, GABA may have a relatively more depolarising effect, which has been attributed to a higher ratio of NKCC1 (*SLC12A2*) to KCC2 activity (*SLC12A5*; (Kaila et al., 2014)). We found that GABA on its own did not trigger any appreciable neuronal activity: the level of cellular activation in response to 1 mm GABA applications was significantly smaller than those to glutamate and similar to the application of aCSF without drugs (a control for any pressure pulse effect; GLU = 68.7 ± 12.0, GABA = 2.42 ± 0.958, aCSF = 0.798 ± 0.427, *p* < 0.001, Kruskal‐Wallis with Dunn's multiple comparison test; Figure 8(a)); the neuropil responses followed the same trend (GLU = 22.6 ± 6.13, GABA = 0.677 ± 0.158, aCSF = 0.485 ± 0.120, *p* < 0.001, Kruskal‐Wallis with Dunn's multiple comparison test; Figure 8(b)).

**FIGURE 8:**
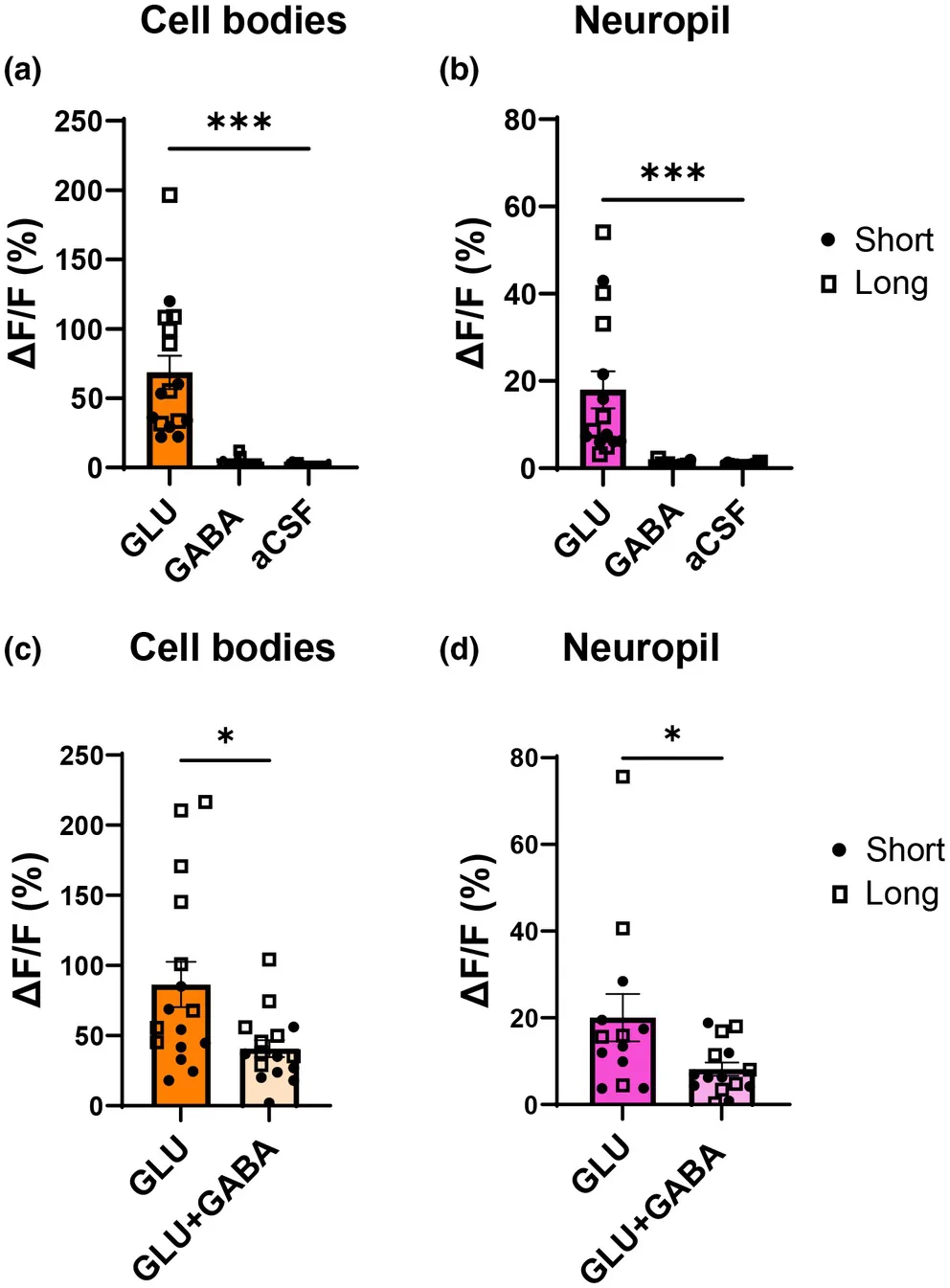
Glutamate evokes calcium transients that are reduced by GABA in human organotypic slice cultures. (a, b) The calcium response (ΔF/F %) to 1 mm glutamate (GLU) or 1 mm GABA was measured in the region of the slice where the highest calcium response to glutamate was detected in human organotypic slice cultures. The mean fluorescence of selected cell bodies was calculated, and glutamate, but not GABA, induces a significantly increased calcium response (*p* < 0.001) compared to aCSF (a). The neuropil response follows the same trend, with glutamate delivery inducing a significantly increased calcium response (*p* < 0.001) compared to aCSF (b). (c, d) The calcium response (ΔF/F %) to 0.5 mm glutamate and 0.5 mm glutamate and GABA combined was measured. The mean fluorescence of selected cell bodies was calculated for both conditions, with glutamate and GABA combined inducing a significantly decreased calcium response (*p* < 0.05) compared to glutamate alone (c). The neuropil response followed the same trend, with the addition of GABA significantly reducing the response (*p* < 0.05) to glutamate (d). Slices were tested either at the short (8–13 DIV, data points appear as filled circles) or long cultures (41–42 DIV, data points appear as empty squares). All data presented as scatter plots displaying slice data points with mean ± SEM and *n* = 8 slices per DIV group from two (16 and 18 pcw) human donors. Statistical significance assessed by Kruskal‐Wallis with Dunn's multiple comparisons for panels a and b, and by Mann–Whitney tests for panels c and d.

Since GABA alone did not induce an excitatory response, we then combined glutamate and GABA (0.5 mm of each in aCSF) to investigate whether the latter shows inhibitory effects. Application of the two neurotransmitters together elicited a somatic response that was half that to application of 0.5 mm glutamate alone (somatic ΔF/F: GLU = 86.5 ± 16.1, GLU + GABA = 40.7 ± 6.08, *p* = 0.0234, Mann Whitney test; Figure 8(c)) and 3‐fold reduced in the neuropil (GLU = 33.4 ± 8.51, GLU + GABA = 10.6 ± 2.86, *p* = 0.0413, Mann Whitney test; Figure 8(d)). We concluded therefore that there were functional glutamatergic and GABAergic responses, but that the GABAergic effects appear to be inhibitory, consistent with previous patch clamp data recorded from human SP neurons (McLeod et al., 2023; Moore et al., 2011). The frequency and strength of spontaneous neuronal activity was additionally modulated by gap‐junctions which serve to coordinate firing in neurons.

### 3.5 Synaptic markers show high expression within the subplate

Prompted by the cross‐laminae differences in evoked calcium responses, we used immunohistochemical labelling to examine the expression of three representative post‐synaptic proteins, the glutamatergic post‐synaptic scaffolding protein HOMER1 (Figure 9(a), (b)), the NMDA receptor subunit NR1/GluN1 coded by *GRIN1* (Figure 9(c), (d)), and the AMPA receptor subunit GluA2 coded by *GRIA2* (Figure 9(e), (f)), at the 3 time points (fixed at 0 DIV, 8–13 DIV (‘short’ duration culture), or 41–42 DIV (‘long’); data collected from 6 to 8 slices per DIV group from two (16 and 18 pcw) human donors). All three markers showed highly significant variation across laminae at all time points, with a broadly consistent pattern; the highest expression was found in the SP. Note that for some comparisons, such as for NR1 labelling in 0 DIV cultures, the mean density of immunoreactivity of a marker was highest in the IZ, although this was not significantly different from the SP, and the lowest in the CP (in every instance, the CP density was lowest, although not every comparison showed a significant difference). The pattern of synaptic marker expression was largely preserved over time in vitro, with the exceptions being that the labelling density of both HOMER1 and GluA2 was significantly increased in the iSVZ/VZ long duration cultures. A consequence of these parallel development expression patterns is that Homer1 expression was tightly correlated with both NR1 (Figure 10(a)) and GluA2 (Figure 10(b)) across the entire data set. A multivariate analysis of the patterns of these three immunolabels indicated that the HOMER1 and GluA2 puncta count/cell were both strongly dependent on the culturing time (HOMER1, *p* = 0.042; GluA2, *p* = 0.002) and the anatomical location. In contrast, NR1 puncta count/cell appears to depend primarily upon the anatomical location but did not show a significant relationship to the culturing time (*p* = 0.12). Interestingly, the pattern of labelling density across the laminae appeared to be well matched to the intensity of the Ca^2+^ transient evoked by focal glutamate application (Figure 10(c)).

**FIGURE 9:**
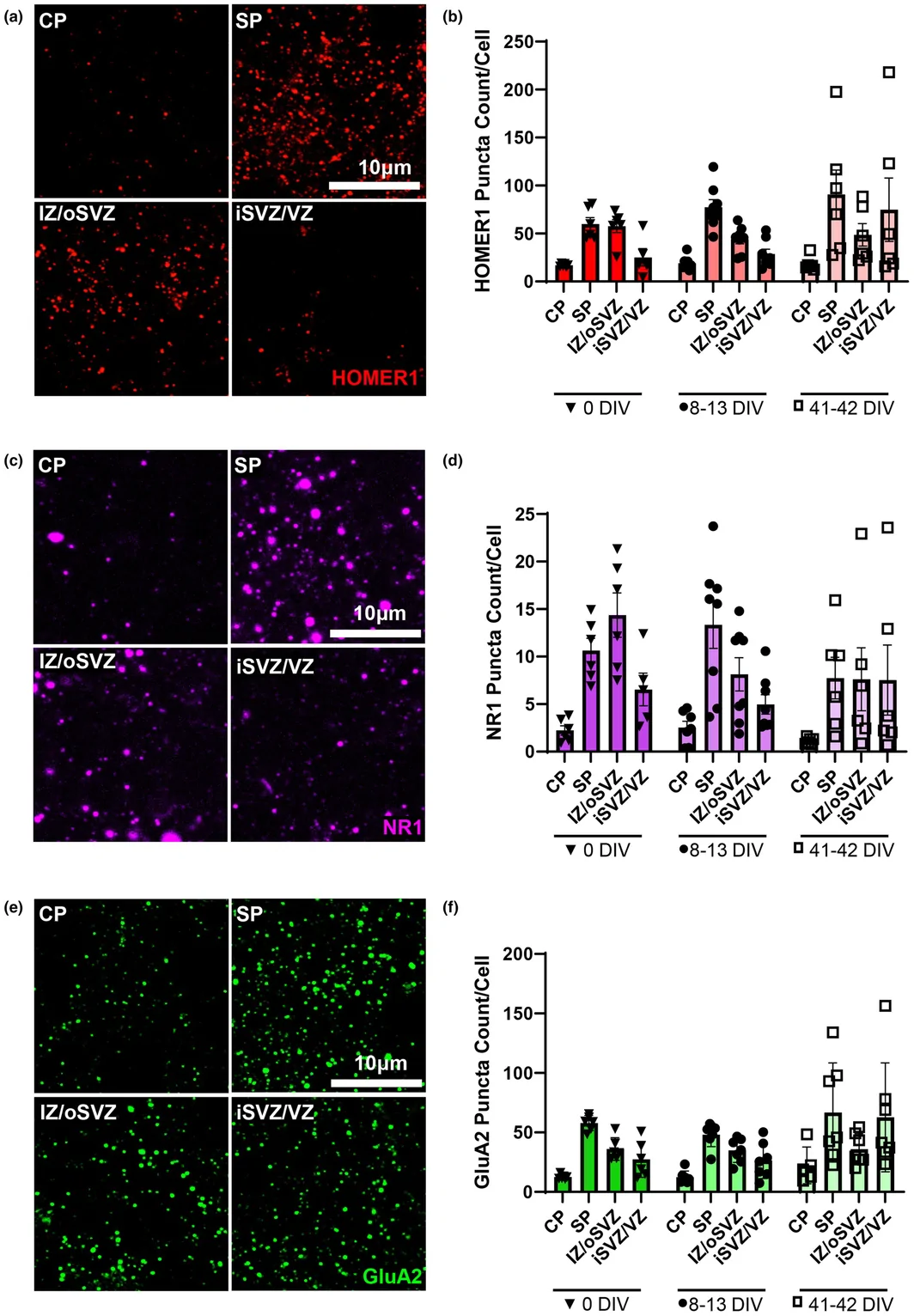
Post‐synaptic markers HOMER1, NR1, and GluA2 are more abundant in the subplate. (a) Representative images from the cortical plate (CP), subplate (SP), intermediate/outer subventricular zone (IZ/oSVZ), and inner subventricular/ventricular zone (iSVZ/VZ) showing labelling for the post‐synaptic scaffolding protein HOMER1. (b) Quantification of HOMER1‐immunoreactive puncta (magenta), expressed as puncta count normalised to cell number across cortical laminae. CP–CP: No significant changes between different time points in culture. CP–SP: Higher puncta count in the SP for 0 DIV (*p* < 0.01), 8–13 DIV (*p* < 0.001), and 41–42 DIV (*p* < 0.001). CP–IZ/oSVZ and CP–iSVZ/VZ: Higher counts only at 0 DIV (*p* < 0.05) and 41–42 DIV (*p* < 0.001) for IZ/oSVZ and iSVZ/VZ respectively. SP–SP: No significant changes between different time points. SP–IZ/oSVZ: Higher counts in SP at 8–13 DIV and 41–42 DIV (*p* < 0.05 for both). SP–iSVZ/VZ: Higher counts in SP at 0 DIV (*p* < 0.05) and 8–13 DIV (*p* < 0.001). IZ/oSVZ–IZ/oSVZ and IZ/oSVZ–iSVZ/VZ: No differences over time. iSVZ/VZ–iSVZ/VZ: Increased counts at 0 DIV vs. 8–13 DIV (*p* < 0.05) and 8–13 DIV vs. 41–42 DIV (*p* < 0.05). (c) Representative images of cortical laminae (as in A) labelled for the NMDA receptor subunit NR1. (d) NR1‐immunoreactive puncta (magenta) quantified as puncta count per cell across cortical laminae. CP–CP: No significant changes between different time points. CP–SP: Higher puncta count in the SP for 0 DIV (*p* < 0.01), 8–13 DIV (*p* < 0.001), and 41–42 DIV (*p* < 0.05). CP–IZ/oSVZ: Higher counts for the IZ/oSVZ at 0 DIV (*p* < 0.001), 8–13 DIV (*p* < 0.05), and 41–42 DIV (*p* < 0.05). CP–iSVZ/VZ: Higher counts only at 41–42 DIV (*p* < 0.05). SP–SP: No significant changes between different time points. SP–IZ/oSVZ and SP–iSVZ/VZ: Higher puncta count only in the SP at 8–13 DIV (*p* < 0.05 and *p* < 0.001, respectively). IZ/oSVZ–IZ/oSVZ: No significant changes between different time points. IZ/oSVZ–iSVZ/VZ: Higher counts in the IZ/oSVZ only at 0 DIV (*p* < 0.01). iSVZ/VZ–iSVZ/VZ: No significant changes between different time points. (e) Representative labelling for the AMPA receptor subunit GluA2 across cortical laminae (as in a). (f) Quantification of GluA2 immunoreactive puncta (green) count per cell across cortical laminae. CP–CP: No significant changes between different time points. CP–SP: Higher puncta count in the SP for 0 DIV (*p* < 0.001), 8–13 DIV (*p* < 0.001), and 41–42 DIV (*p* < 0.001). CP–IZ/oSVZ: No differences. CP–iSVZ/VZ: Higher counts in the iSVZ/VZ only at 41–42 DIV (*p* < 0.001). SP–SP: No significant changes between different time points. SP–IZ/oSVZ and SP–iSVZ/VZ: Higher puncta count in the SP at 41–42 DIV (*p* < 0.01) and 0 DIV (*p* < 0.05), respectively. IZ/oSVZ–IZ/oSVZ: No significant changes different time points. IZ/oSVZ–iSVZ/VZ: Higher counts in the iSVZ/VZ only at 41–42 DIV (*p* < 0.05). iSVZ/VZ–iSVZ/VZ: Increased counts at 0 DIV vs. 8–13 DIV (*p* < 0.05) and 8–13 DIV vs. 41–42 DIV (*p* < 0.01). All data presented as scatter plots displaying slice data points with mean ± SEM and *n* = 6–8 slices per DIV group from two (16 and 18 pcw) human donors. Statistical significance assessed by Mixed‐effects model with Tukey's multiple comparisons test.

**FIGURE 10:**
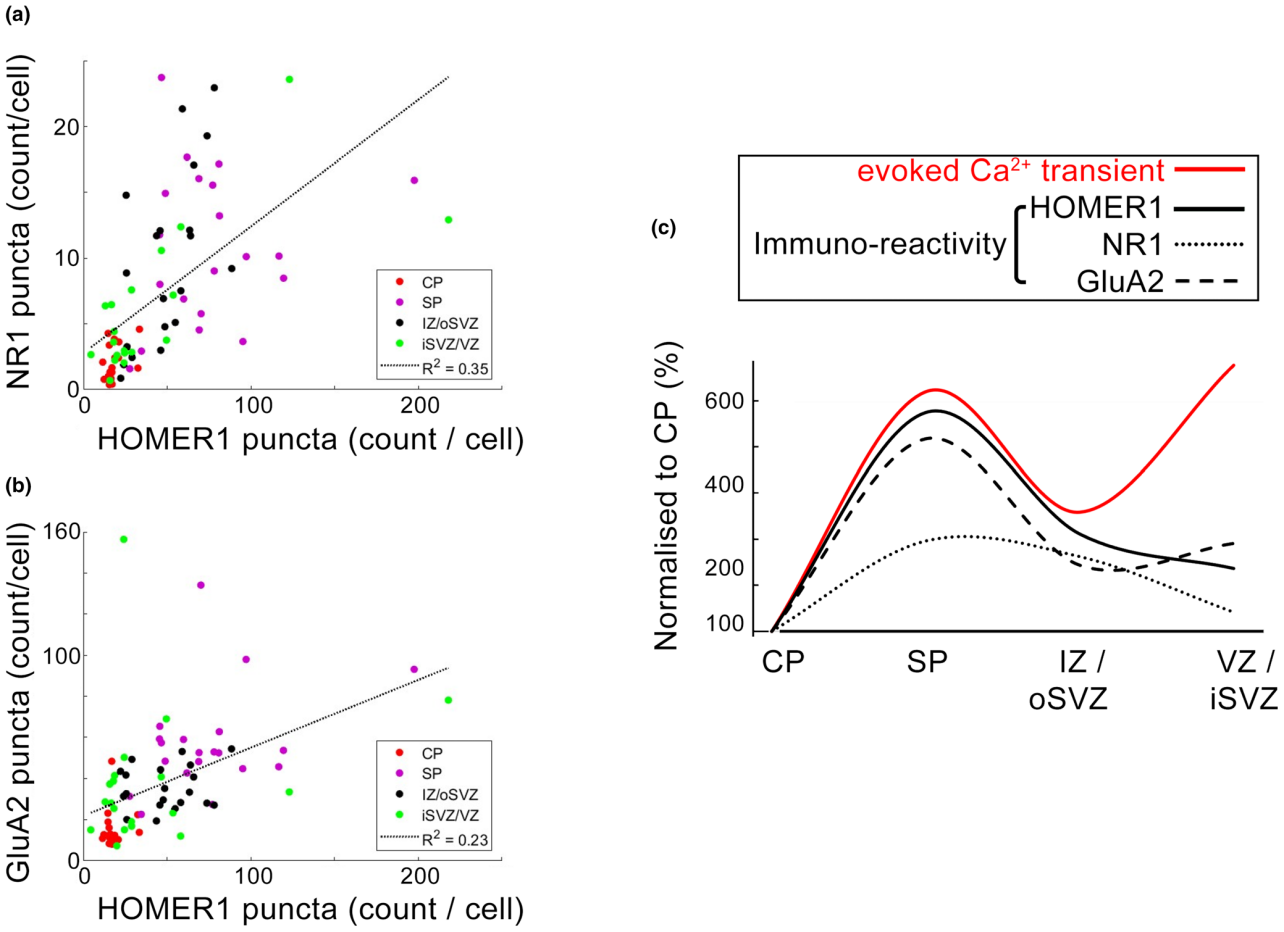
Correlated immunoreactivity and Ca^2+^ imaging data sets. (a, b) Regression analysis shows a highly significant positive association between HOMER1 and NR1 (*R* ^2^ = 0.35, *p* < 0.001, panel a) and between HOMER1 and GluA2 (*R* ^2^ = 0.23, *p* < 0.001, panel b). Data of puncta count/cell from all culturing time point groups and regions were analysed. Data points appear in red for the cortical plate (CP), in magenta for the subplate (SP), in black for the intermediate/outer subventricular zone (IZ/oSVZ), and in light green for the inner subventricular/ventricular zone (iSVZ/VZ). (c) The same immunoreactivity data from the different cortical areas are plotted normalised to the level of expression in the cortical plate (black lines), together with the amplitude of the evoked Ca^2+^ responses (data in Figure 7) normalised in the same way. Note how the regional variation in the amplitude of the glutamatergic response mirrors the change in expression of both GluA2 and HOMER1 expression. CP, cortical plate; iSVZ, inner subventricular zone; IZ/oSVZ, intermediate/outer subventricular zone; SP, subplate. *N* = 6–8 slices per DIV group from two (16 and 18 pcw) human donors.

## 4 DISCUSSION

In this study, we extended the characterisation of our experimental platform for examining prenatal human neocortical development. Building upon our prior descriptions (McLeod et al., 2023, 2026), here we examined the evolving patterns of gene expression changes in our organotypic cultures and further demonstrated the existence of functional chemical and electrical synaptic communication between a morphologically diverse population of neurons. Notably, the expression of various synaptic proteins appeared well matched to the pattern of responses to glutamatergic stimuli, both being highest in the SP and SVZ (Figure 10(c)).

The transcriptomic data identified clear differences in gene expression between the day of preparation of organotypic cultures (0 DIV) and 14 days later, but this needs to be considered also in the context of ongoing developmental changes. The ethical approval for collection of human brain tissue was restricted to before 18 pcw. Our organotypic cultures were prepared from pcw 16–18, and so by 14 DIV, their biological age exceeded that of any of the fresh samples. Instead, we compared the changes in culture from 0 to 14 DIV with extrapolations from tissue RNAseq data collected previously from 7.5–17 pcw occipital cortex (all could be considered as “DIV 0”). This prior RNAseq data was collected using a different experimental platform (Illumina HiSeq 2000; (Lindsay et al., 2016)), but normalization to reference genes gave a good correspondence with our NanoString 0 DIV. This provided some validation of the comparisons between 0 and 14 DIV, by highlighting that the genes that showed greatest upregulation generally did so from very low levels of expression in 0 DIV samples. In contrast, many synaptic genes showed changes between 0 and 14 DIV, but these followed normal developmental trajectories. Some gene expression changes could be explained by the presence of growth factors in the culture media. For example, WNT7A and WNT7B are highly homologous Wnt ligands sharing 77.1% amino acid identity (Kirikoshi et al., 2001), and have partially redundant biological functions (Cho et al., 2019). Thus, the observed reduction in *WNT7B* expression may reflect a compensatory feedback response to exogenous WNT7A present in the culture media. Although this mechanism has not been directly demonstrated in our culture system, it is consistent with the shared signalling properties of these ligands.

Interestingly, the cluster analyses of the different gene expression changes in the cultures consistently grouped the samples into the three brains. This may reflect some unidentified background genetic differences between the three brains, but it may also result from differences such as post‐mortem interval to culturing, precise age post‐conception, or some other technical detail not exactly replicated in all three cases. It does, however, emphasize the importance of considering sample effects. The differences between the brains were flagged in the 2nd principal component, representing a small proportion of the total variance (10%) across all the samples at the two ages. The much larger, first principal component captured most of the difference between 0 and 14 DIV. In other words, there was a much bigger difference in gene expression between 0 and 14 DIV than there was between the brains, and this was also evident in the heatmaps, where the gene expression changes between 0 and 14 DIV were highly consistent in their direction across all samples.

Some of the gene expression changes suggest that various cells were under stress within cultures. Marker genes for reactive astrocytes and microglia showed upregulation whereas markers for resting microglia were downregulated, suggesting that culturing engenders a neuroinflammatory response. We also observed a small but significant down‐regulation of *TP53* in response to culturing, but two other genes that are immediate downstream targets of p53, *CDKN1A* and *TNFRSF10B*, were among the most highly up‐regulated. Finally, genes for neuroprotective molecules were also upregulated.

Of the downregulated genes, perhaps the most predictable changes were for genes involved in maintaining vascular integrity, given that the removal of a vascular supply would deprive the tissue of any mechano‐transductive signals and bloodborne growth factors that might promote vascular growth and maintenance (Longo & Goyal, 2013). Also noteworthy was the downregulation of *FN1*, which is ordinarily expressed at high levels within the radial glia (Peregrina & Del Toro, 2020). A loss of FN1 might be expected to affect neuronal migration to the CP and could possibly explain why we observed ectopic neuronal responses within the SVZ.

The relative levels of expression of the two cation‐chloride co‐transporters, KCC2 and NKCC1, are thought to be important determinants of intracellular [Cl^−^], which in turn affects a neuron's response to GABA. If the KCC2/NKCC1 ratio is high, Cl^−^ removal is facilitated, leading to lower [Cl^−^]_in_ and relatively hyperpolarising GABA effects. Conversely, a low KCC2/NKCC1 ratio can cause GABA to be depolarising. We were interested therefore in the large drop between 0 and 14 DIV in *SLC12A5* gene expression, which encodes for KCC2. This result contrasts with the upwards trend in expression from 7.5 to 17 pcw during normal development (Figure 5), and our previous observations of strong immunoreactivity for KCC2 in pyramidal‐like cells in the human subplate at 16 pcw (Bayatti et al., 2008), and comparable mRNA expression of *SLC1512A5* and *SLC12A2* (NKCC1) in human foetal cortical tissue (Lindsay et al., 2016). The reduced KCC2 levels might therefore have been expected to create a depolarising GABAergic response, but instead, we found GABA applications to be inhibitory (Figure 7), more in line with the hyperpolarising effect of GABA recorded from subplate neurons in acute slices of mid gestation human cortex (Moore et al., 2011). The inhibitory effect of GABA, in spite of apparently low KCC2 expression in the cultures may be explained in several ways. First, the NanoString panel did not include *SLC12A2*, so it is possible that NKCC1 expression is also greatly reduced in the cultures, thus offsetting the reduction in KCC2 expression. Second GABA_A_ can produce inhibition through shunting effects, even in cases where it is depolarising, especially if the reversal potential still lies below action potential threshold. Alternatively, the inhibitory effect may be mediated by GABA_B_ activation. Consistent with this is that GABA_B2_ and GABA_B3_ receptors show preserved levels of expression in the cultures at 14 DIV.

Our functional studies flagged up two other key results. Firstly, drugs that block gap junctions and hemi‐channels markedly reduced the amplitude of spontaneous Ca^2+^ transients, although these occurred with increased frequency, suggestive that these channels may play a role in coordinating activity across the tissue. A similar conclusion was drawn by Moore et al. (2014), seeing a large drop in subplate neuron firing, induced by a slightly different cocktail of blockers (carbenoxolone, octanol and flufenamic acid), when applied to acutely prepared human foetal brain slices (Moore et al., 2014). Moore et al. recorded single cell activity patterns using patch clamp but even allowing for the different methodology for assaying neuronal firing, the acute and cultured brain slices appear to differ: in the acute preparations, the patched neurons fired continually (Moore et al., 2011, 2014) whereas in the cultured brain slices (Figure 6), we observed large Ca^2+^ transients that were synchronised across neurons, indicative of large, coordinated network discharges. Such coordinated activity can arise from gap‐junction coupling, but not necessarily so. It is noteworthy, therefore, that Moore et al. (2014) concluded that in their acute preparation, the drug effects arise from blocking hemi‐channels, rather than gap‐junctions, based on two pieces of evidence. First was that when neurons were filled with dyes that cross gap‐junctions, they did not observe any dye coupling, and second, that the reduction in neuronal activity was also observed with lanthanum application, which is thought to block only hemi‐channels and not gap‐junctions.

The second point is that activity appeared mostly in the SP area, in line with prior work showing that this is where the earliest synapses form (Hanganu et al., 2002; Kostovic, 2024; Kostovic & Rakic, 1990). This also aligns well with prior in vitro studies showing that CP and SP neurons appear more active than neurons from proliferative zones (Mayer et al., 2019; Moore et al., 2009). GFP neurons were concentrated in the SP region. Expression of the pre‐synaptic markers STXBP1, vGLUT1 and synaptophysin has been shown to be higher in the SP and MZ (Bayatti et al., 2008; Kostovic et al., 2019; McLeod et al., 2023). For postsynaptic markers such as HOMER1, GluA2 (*GRIA2* gene) and NR1, expression is much greater in the SP than CP (Figure 8), but interestingly, the difference between the SP and IZ/SVZ was not so marked suggesting that immature, migrating neurons may express glutamate receptors with glutamate acting as a paracrine or autocrine regulator of migration (Luhmann et al., 2015). However, once settled in the CP, expression is downregulated until the neurons later become integrated into synaptic circuits, which occurs after mid‐gestation (Kostovic, 2024).

In summary, we suggest, therefore, that organotypic cultures prepared from human foetal brains represent a valid means of addressing research questions about the early period of human cortical development that occurs prenatally, which are otherwise beyond our scope. These cultures preserve key aspects of cortical architecture, with diverse populations of differentiated neurons that show spontaneous firing activity and make functional synaptic connections. On the other hand, our gene expression analyses suggest that organotypic cultures may diverge from the normal developmental programme in particular ways, with evidence of culture‐associated cellular stress and changes that might be associated with deafferentation and devascularization. These all should be considered carefully when interpreting the downstream effects of any experimental manipulation, but the possibilities for studying such manipulations remain the most important benefit of this preparation.

## FUNDING INFORMATION

This research was funded by an Emerging Leader Fellowship Award to McLeod (F2202) and PhD studentship to Dimtsi (DTC2101), both awarded by Epilepsy Research Institute UK. For open access, the author has applied a CC BY public copyright licence to any Author Accepted Manuscript version arising from this submission. The work of the HDBR was funded by the Wellcome Trust and the Medical Research Council (MR/R006237/1).

## CONFLICT OF INTEREST STATEMENT

The authors report no competing interests.

## Data Availability

The data that support the findings of this study are available on request from the corresponding author. The data are not publicly available due to privacy or ethical restrictions.
